# Climate-driven redistribution of foxtail millet (*Setaria italica*) suitability in China revealed by biomod2 ensemble models: implications for dryland crop adaptation

**DOI:** 10.3389/fpls.2026.1882902

**Published:** 2026-09-09

**Authors:** Lina Chang, Jiakun Yan, Xiaolin Wang, Xiong Zhang, Yuming Chang, Ke Lu

**Affiliations:** 1 Shaanxi Key Laboratory of Ecological Restoration in Northern Shaanxi Mining Area, China College of Advanced Agricultural Sciences, Yulin University, Yulin, China; 2 First Clinical Medical College, College of Information and Engineering, Wenzhou Medical University, Wenzhou, China

**Keywords:** biomod2, centroid migration, climate change, dryland agriculture, habitat suitability, *Setaria italica*, species distribution model

## Abstract

Foxtail Millet (*Setaria italica*) is one of China’s earliest domesticated cereal crops and remains important for dryland farming, germplasm conservation, and climate-resilient agriculture. However, climate warming and altered precipitation regimes may reshape its agroclimatic suitability and challenge traditional production regions. Here, we integrated 1, 948 cleaned occurrence records with climatic, topographic, soil, and UV-B radiation variables, and used the biomod2 ensemble species distribution modelling framework to predict the potential suitable distribution of *S. italica* across China. Projections were conducted under the historical baseline and CMIP6 BCC-CSM2-MR future climate scenarios for SSP1-2.6, SSP2-4.5, SSP3-7.0, and SSP5-8.5 across the 2030s, 2050s, 2070s, and 2090s. The selected EMwmeanByTSS ensemble showed good discrimination ability and acceptable predictive performance, with validation ROC and TSS values of 0.863 ± 0.011 and 0.581 ± 0.022, respectively. Annual precipitation (bio12, 29.61%), elevation (elev, 28.07%), minimum temperature of the coldest month (bio06, 14.23%), and UV-B seasonality (uvb2, 10.93%) were the dominant predictors, jointly contributing 82.82%. Under the historical baseline, the total potential suitable area was 4.09 × 10^6^ km² (44.22% of China’s land area), with highly suitable areas concentrated in the North China Plain, eastern Loess Plateau, and southern margin of Northeast China. Under future SSP scenarios, the total suitable area increased to 4.29 × 10^6^ – 4.87 × 10^6^ km², representing a 4.77%–18.87% increase. Expansion was mainly projected along the northern and peripheral margins of the current suitable region, while contraction occurred along southern, southwestern, and transitional margins. The suitability centroid shifted slightly northward to northwestward from northern Henan toward southern–central Shanxi, with net displacement of 113.33–232.79 km by the 2090s. Overall, future suitability is projected to show a pattern of stable core areas, marginal expansion, localized contraction, and limited centroid migration. These findings provide a spatial basis for stable production-region protection, regional variety trials, germplasm conservation, and climate-adaptive dryland agricultural planning.

## 1 Introduction

Foxtail Millet (*Setaria italica*) is among the earliest domesticated cereal crops and remains a representative grain crop in traditional dryland farming systems of northern China (Wheeler and Von Braun, 2013). Archaeological evidence indicates that millets were central to the emergence and development of early agriculture in northern China, especially across the Yellow River Basin, the North China Plain, and adjacent regions (Crawford, 2009; Lu et al., 2009; Yang et al., 2012). Compared with major staple crops such as rice, wheat, and maize, *S. italica* has a shorter growth duration, stronger drought tolerance, greater adaptation to infertile soils, and relatively high water-use efficiency, making it particularly valuable in arid, semi-arid, and monsoon-margin agroecosystems (Lata et al., 2013; Yongqing et al., 2014). In recent years, *S. italica* has also been recognized as a model system for studying C4 photosynthesis, grass evolution, stress adaptation, and cereal improvement because of its small genome, short life cycle, and close relationship with related Panicoid grasses (Li and Brutnell, 2011; Bennetzen et al., 2012; Zhang et al., 2012). However, dryland agricultural systems in northern China are increasingly exposed to climate extremes, including intensified drought events, heatwaves, and compound hot–dry conditions. Recent studies have demonstrated that these extreme events can substantially affect crop growth, yield stability, and agricultural sustainability in northern China, particularly in water-limited regions (Zhang et al., 2022; Zhu et al., 2022; Xu et al., 2024).

Under intensifying climate change and increasing agricultural water constraints, reassessing the potential suitable distribution of *S. italica* and its future spatial reorganization is important for optimizing dryland cropping systems, conserving regional germplasm resources, and improving agricultural resilience (Wheeler and Von Braun, 2013; Challinor et al., 2014; Altieri et al., 2015). Climate change is reshaping the geography of crop suitability (Lobell et al., 2011). Warming may extend the growing season in some high-latitude or cool regions and promote poleward or upslope shifts of suitable conditions for temperate crops (Rosenzweig et al., 2014). At the same time, altered precipitation regimes, increasing drought frequency, humid-heat stress, and more frequent extreme events may cause local contraction or suitability-class redistribution within existing production regions (Lobell et al., 2011; O’Neill et al., 2016; Jägermeyr et al., 2021). For a typical dryland crop such as foxtail millet, suitability cannot be interpreted simply as increasing under drier or warmer conditions (Huang et al., 2023). Although the crop is drought tolerant and water efficient, stable growth still requires appropriate water supply, thermal conditions, and growing-season length; excessive aridity, humid-heat conditions, high-elevation cold limitation, and seasonal mismatch can all constrain its potential distribution (Lata et al., 2013; Liu et al., 2020). Thus, the formation and future redistribution of *S. italica* suitability should be understood as the combined outcome of moisture availability, temperature limitation, topographic gradients, soil background, radiation conditions, and agroecological context.

Species distribution models (SDMs) provide a statistical framework for linking species occurrence records with environmental predictors and projecting potential suitable areas under current and future climate conditions (Guisan and Zimmermann, 2000; Elith and Leathwick, 2009). SDMs have been widely applied in crop suitability assessment, climate adaptation studies, invasion risk evaluation, and conservation planning (Hijmans and Graham, 2006; Phillips et al., 2006; Booth et al., 2014). However, predictions generated from individual algorithms may vary substantially because of differences in model structure, parameterization, background selection, and calibration procedures. Ensemble SDMs integrate multiple algorithms and reduce dependence on a single modelling framework, thereby improving prediction stability and reducing algorithm-related uncertainty (Araujo and New, 2007; Marmion et al., 2009; Thuiller et al., 2009). The biomod2 platform provides an effective framework for multi-algorithm comparison, ensemble prediction, variable contribution analysis, response curve interpretation, and future suitability projection (Thuiller et al., 2009).

Although SDMs have been increasingly applied to crop-climate assessments, current knowledge regarding the potential distribution of *S. italica* in China remains incomplete. Previous studies have mainly relied on individual algorithms for suitability prediction, while uncertainty associated with different modelling approaches has rarely been systematically evaluated (Elith et al., 2006; Phillips et al., 2006; Araujo and New, 2007a). Moreover, previous assessments have largely focused on describing current suitability patterns, whereas comprehensive evaluations of future suitability redistribution, including suitability-class transitions, spatial expansion and contraction, centroid migration, and nonlinear responses to environmental gradients, remain limited. Such integrated assessments are essential because climate change may alter not only the extent but also the spatial configuration of crop suitability (Lobell et al., 2011). Therefore, a robust ensemble modelling framework is needed to provide more reliable projections and identify regions requiring future field validation and adaptive management (Wang et al., 2010; Cowan et al., 2013; Jia et al., 2013; Pan et al., 2015).

Accordingly, this study developed an ensemble SDM framework by integrating occurrence records of *S. italica* with climatic, topographic, soil, and UV-B radiation variables. Specifically, this study aimed to: (1) compare the predictive performance of multiple single algorithms and ensemble models, and identify dominant environmental drivers and their nonlinear relationships with suitability; (2) characterize current suitability patterns and project future changes under multiple CMIP6 SSP scenarios; and (3) quantify future expansion, contraction, and centroid migration of suitable areas to provide spatial insights for climate-adaptive cultivation planning and germplasm conservation.

## 2 Materials and methods

### 2.1 Occurrence data collection and processing

Occurrence records of *S. italica* across China were compiled from local floras, crop-resource surveys, published literature, digital herbaria, and germplasm databases, including the Chinese Virtual Herbarium, the Global Biodiversity Information Facility (GBIF, http://www.gbif.org, accessed on 20 February 2026), the Chinese Crop Germplasm Resources Information System, the Teaching Herbarium Standardization and Resource Sharing Platform, and the China Nature Reserve Resource Platform. Approximately 5, 000 raw occurrence records were initially collected, broadly covering the current known distribution of *S. italica* in China.

**Figure 1:**
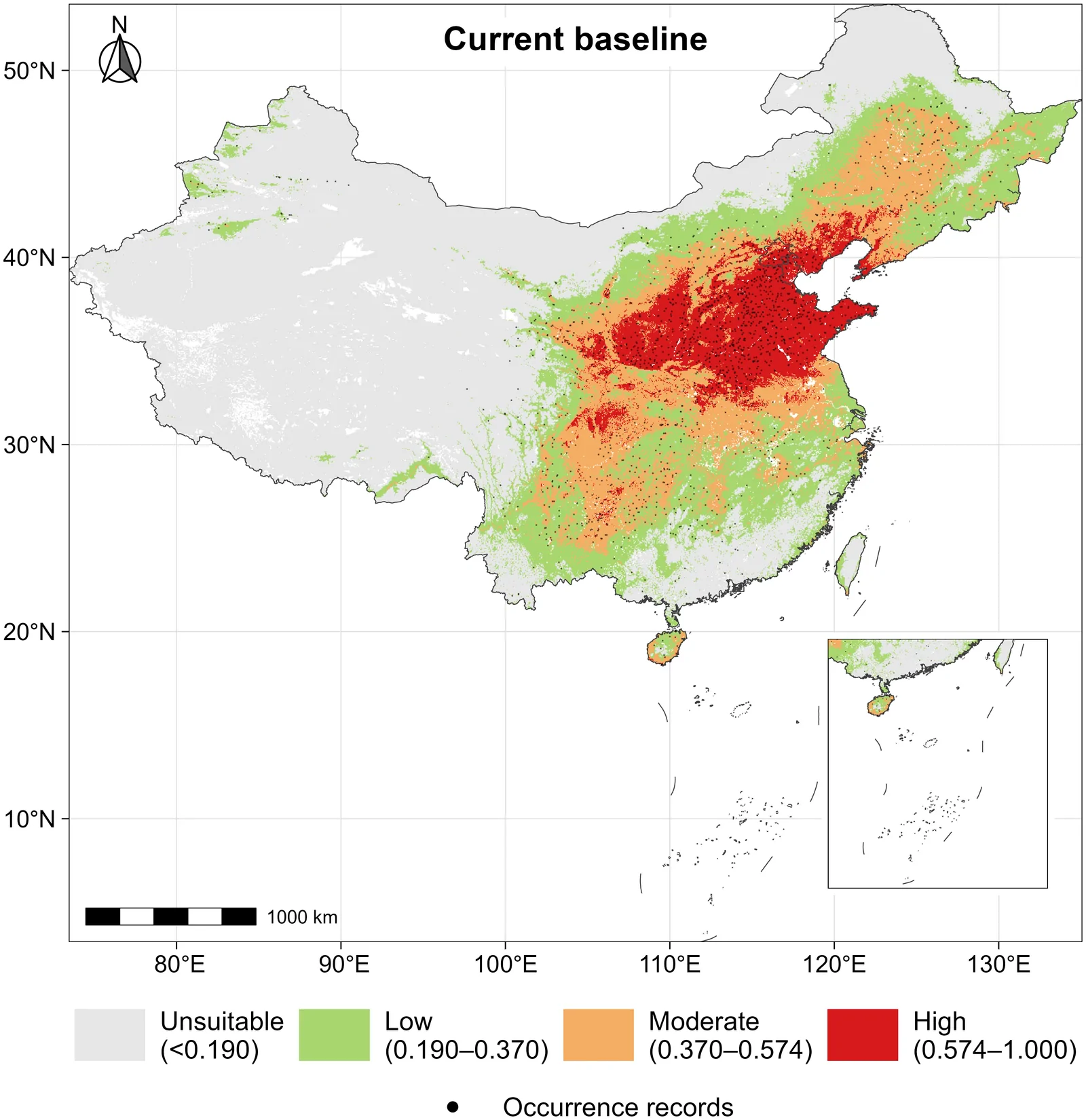
The current climate-based distribution prediction and occurrence records distribution of *Setaria Italica*.

To improve data reliability and spatial representativeness, all raw records were screened, standardized, and georeferenced. Records with ambiguous taxonomic identity, missing coordinates, incomplete locality descriptions, or exact duplicates were removed. For records with detailed locality information but without coordinates, locality descriptions were reconstructed using specimen metadata and relevant literature, and coordinates were checked using Google Earth. Spatial quality control was then performed using ArcGIS 10.4 and the CoordinateCleaner package in R 4.2.3 to remove records falling outside the terrestrial boundary of China, in oceans or water bodies, with swapped longitude/latitude, zero coordinates, administrative-centre coordinates, institutional default coordinates, or other obvious geocoding errors (Zizka et al., 2019). To reduce spatial clustering and sampling bias while retaining broad distributional coverage, records were spatially thinned using ENMTools, retaining one occurrence within each 5 km x 5 km grid cell (Warren et al., 2010). After these procedures, 1, 948 valid occurrence records were retained for model construction (**Figure 1**; **Supplementary Table 1**).

### 2.2 Environmental variables and multicollinearity control

The environmental framework included a historical climate baseline and future climate scenarios. The baseline climate represented mean conditions during 1970-2000, and future periods were defined as the 2030s (2021–2040), 2050s (2041–2060), 2070s (2061–2080), and 2090s (2081–2100). Initial candidate variables included climatic, topographic, soil, and UV-B radiation variables. Bioclimatic (bio1-bio19) and topographic variables (elev/aspect/slope) were obtained from WorldClim v2.1 (http://www.worldclim.org/) (Fick and Hijmans, 2017); soil variables were obtained from the Harmonized World Soil Database (HWSD, https://www.fao.org/soils-portal/en/); and UV-B radiation variables (UVB1-4) were obtained from the gIUV-B dataset for macroecological studies (http://www.ufz.de/gluv/) (Bennetzen et al., 2012). All environmental layers were resampled to a spatial resolution of 2.5 arc-minutes (approximately 5 km) and clipped to the terrestrial area of China (**Supplementary Table 2**). For future projections, bioclimatic variables were replaced with corresponding CMIP6-derived climate projections under different SSP scenarios, whereas topographic variables (elevation, aspect, and slope), soil variables, and UV-B radiation variables remained unchanged.

**Figure 2:**
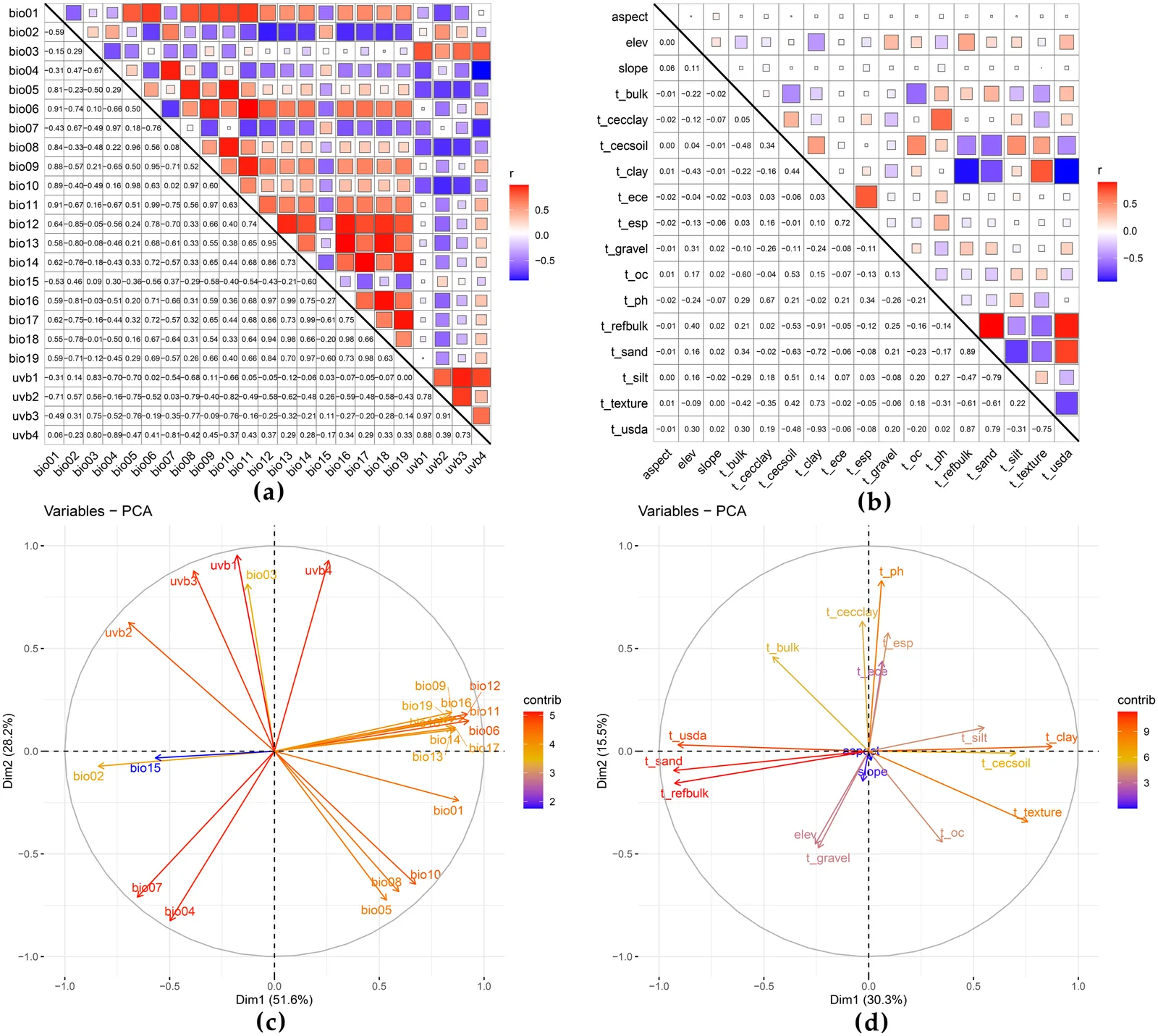
Selection of environmental variable correlations. **(a, b)** respectively represent the correlations between climate and soil topography environmental variables. **(c, d)** respectively represent the PCA screening of climate and soil topography environ-mental variables.

Future climate projections were based on the BCC-CSM2-MR global climate model under the CMIP6 framework. Four Shared Socioeconomic Pathways (SSPs) were used: SSP1-2.6, SSP2-4.5, SSP3-7.0, and SSP5-8.5, representing low, medium-low, medium-high, and high emission pathways, respectively (Eyring et al., 2016; O’Neill et al., 2016). In future projections, topographic and soil variables were kept constant, whereas climate and UV-B variables were replaced by scenario- and period-specific layers. To reduce multicollinearity and improve model interpretability, Pearson correlations among candidate variables were calculated. When a pair of variables showed |r| ≥0.8, one variable was retained based on ecological relevance, relationship with *S. italica* distribution, preliminary contribution, and interpretability (Dormann et al., 2013; Merow et al., 2013). Principal component analysis (PCA) was further used to assess the distribution and redundancy of candidate variables in environmental-gradient space and to verify whether retained variables covered major axes of environmental variation. Based on Pearson correlations, PCA ordination, ecological interpretability, and preliminary model contribution, 12 environmental variables were retained for modelling (**Figure 2**; **Table 1**).

**Table 1:** Explanation of environmental variables.

Environment variable	Description	Unit
Bio4	Seasonal variation coefficient of temperature (standard deviation×100)	-
Bio5	Max Temperature of Warmest Month	
Bio6	Min Temperature of Coldest Month	°C
Bio12	Annual Precipitation	mm
Bio15	Precipitation Seasonality	mm
t_cecsoil	Topsoil cation exchange capacity of the soil	kg^−1^ soil
S_Sand	Soil Sand	%
t_oc	Topsoil organic carbon	% weight
t_ph	Topsoil pH	-
UVB1	Annual Mean UV-B	J/m^2^/day
UVB2	UV-B Seasonality	J/m^2^/day
DEM	Digital Elevation Model	m
Slope	Slope	°

temperature seasonality (bio4) given their nature as ratios or standardized scales, they are presented without physical units in this table.

### 2.3 Species distribution modelling and evaluation

Species distribution modelling was conducted in R 4.2.3 using the biomod2 platform (Thuiller et al., 2009). To reduce dependence on a single model structure, eight algorithms were implemented: classification tree analysis (CTA), flexible discriminant analysis (FDA), generalized linear model (GLM), generalized boosting model (GBM), random forest (RF), maximum entropy (MAXENT), multivariate adaptive regression splines (MARS), and eXtreme Gradient Boosting (XGBOOST). For each algorithm, 75% of occurrence records were randomly used for calibration and 25% for validation, with three independent replicate runs (Fielding and Bell, 1997). Because true absence data were unavailable, 6, 000 pseudo-absence/background points were randomly generated within the terrestrial area of China and used together with occurrence records for model construction.

Before formal modelling, the ENMeval package was used to optimize MaxEnt parameters, including the regularization multiplier (RM) and feature classes (FC) (Muscarella et al., 2014). The evaluated feature classes included linear (L), quadratic (Q), hinge (H), product (P), and threshold (T) features. RM values ranged from 0.5 to 4.0 at intervals of 0.5, and six FC combinations were tested: L, LQ, H, LQH, LQHP, and LQHPT, resulting in 48 candidate parameter combinations. The optimal combination was selected using corrected Akaike information criterion (AICc), with differences between training and testing AUC and the 10% test omission rate used to assess potential overfitting (Muscarella et al., 2014; Phillips et al., 2017). The optimized MaxEnt settings were used in subsequent biomod2 modelling, whereas the remaining algorithms used biomod2 default settings. Complete MaxEnt optimization results are provided in **Supplementary Table 3**.

Model performance was evaluated using ROC, TSS, and KAPPA (Allouche et al., 2006; Liu et al., 2013). Ensemble models were constructed using three strategies: EMmean (unweighted mean probability), EMca (committee averaging), and EMwmean (performance-weighted mean probability). To reduce the influence of weak algorithms, the top five single models were retained for ensemble prediction. Considering validation results, continuous suitability output, and the requirements of suitability classification, area statistics, range-change analysis, and centroid migration, EMwmeanByTSS was selected as the final prediction model. Environmental variable importance was extracted from the final ensemble model, and response curves were generated to characterize changes in predicted suitability along environmental gradients. Curve segments near peak suitability or exceeding a predicted suitability value of 0.70 were used to identify high-suitability response intervals; this value served only as an interpretative criterion for describing modelled response patterns rather than an absolute ecological threshold.

### 2.4 Suitability classification and area statistics

The final model produced continuous suitability values ranging from 0 to 1, with higher values indicating greater predicted suitability. For spatial visualization, area statistics, and scenario comparisons, continuous EMwmeanByTSS outputs were classified using the Jenks natural breaks method. Four suitability classes were defined: unsuitable (<0.190), low suitability (0.190–0.370), moderate suitability (0.370–0.574), and high suitability (0.574–1.000). These classes were used only for relative spatial classification and comparison among regions and scenarios. They represent model-based suitability categories rather than absolute physiological or ecological thresholds for *S. italica.* To evaluate the performance of the binary threshold (0.190), predicted suitability values were extracted from the final EMwmeanByTSS model at all occurrence locations. Occurrence records located in NoData areas were excluded, and the omission rate was calculated as the proportion of valid occurrence records with predicted suitability values below the threshold (Zhao et al., 2021).

### 2.5 Range-change and centroid migration analyses

To assess future spatial changes, binary suitable/unsuitable maps for the baseline and future periods were overlaid in ArcGIS using SDMtoolbox. Four range-change categories were identified: stable unsuitable area, stable suitable area, expansion area, and contraction area. Stable unsuitable area referred to regions unsuitable in both baseline and future periods; stable suitable area referred to regions suitable in both periods; expansion area referred to baseline-unsuitable regions projected to become suitable; and contraction area referred to baseline-suitable regions projected to become unsuitable. Expansion, contraction, stable suitable, and stable unsuitable areas were quantified for each SSP-period combination, and net change was calculated as expansion area minus contraction area (Zhao et al., 2021). Centroid migration was also analysed using binary suitable areas. The geographical centroid of the suitable area was calculated for the baseline and each future SSP-period combination using SDMtoolbox. Taking the baseline centroid as the reference, future centroid displacement distance and direction were calculated to summarize potential shifts in the spatial center of *S. italica* suitability.

## 3 Results

### 3.1 Model evaluation and ensemble selection

**Figure 3:**
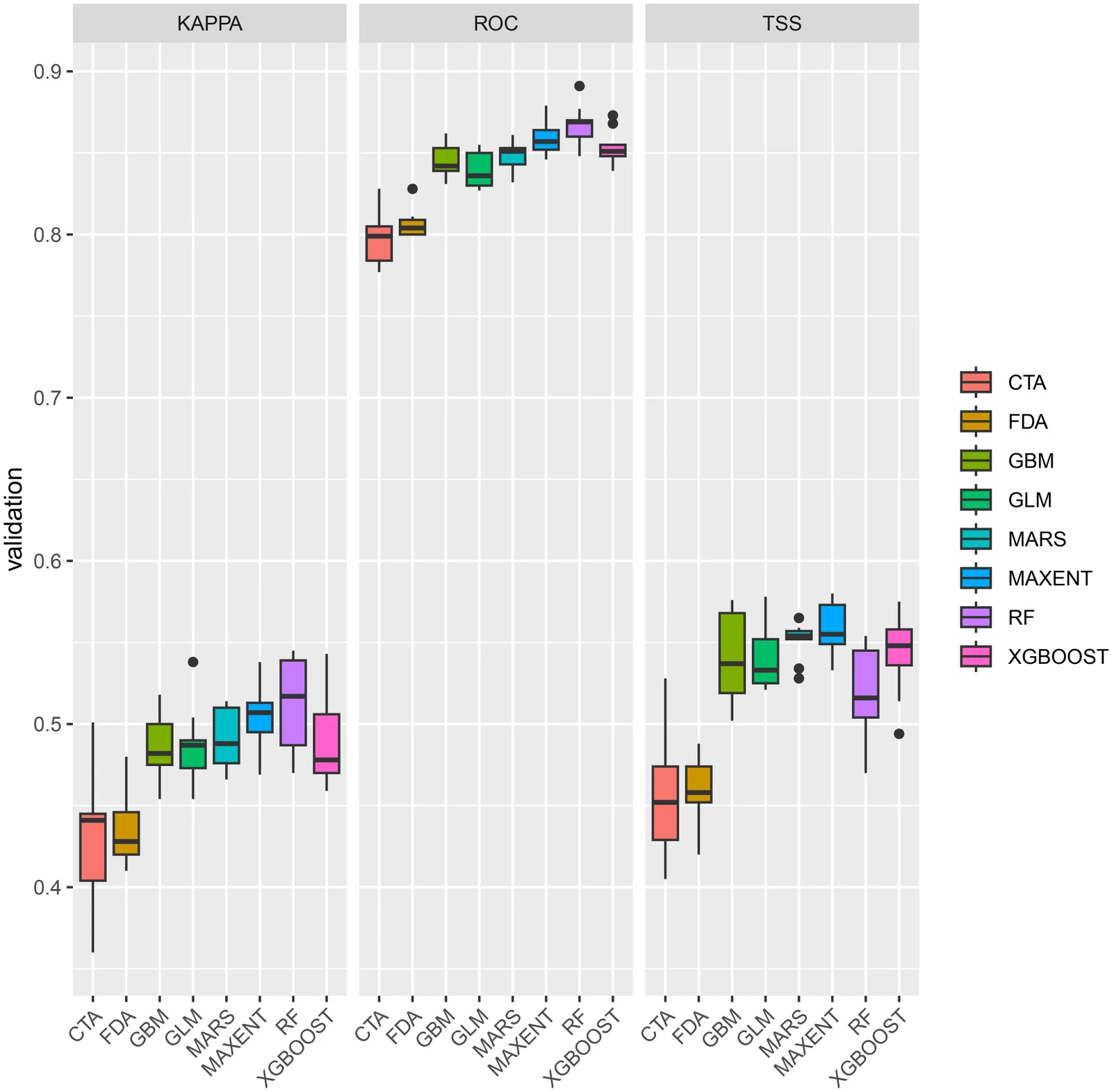
Accuracy of a single algorithm model prediction.

The eight single-algorithm models differed in predictive performance but generally showed acceptable to acceptable discrimination ability for predicting the potential suitable distribution of *S. italica* in China (**Figure 3**; **Supplementary Table 4**). ROC values ranged from0.797 to 0.867, TSS values from0.456 to 0.558, and KAPPA values from0.427 to 0.511. RF showed the highest ROC and KAPPA values, at0.867 ± 0.012 and0.511 ± 0.028, respectively, whereas MAXENT had the highest TSS value (0.558 ± 0.017). MARS, MAXENT, RF, and XGBOOST all had ROC values above0.848, indicating relatively strong discrimination ability. By contrast, CTA and FDA showed lower evaluation metrics, with ROC, TSS, and KAPPA values of 0.797 ± 0.016, 0.456 ± 0.038, and0.427 ± 0.043 for CTA, and0.807 ± 0.00, 0.461 ± 0.020, and 0.435 ± 0.023 for FDA, respectively.

Based on the selected top five single-algorithm models, three ensemble strategies were further constructed: EMmean, EMca, and EMwmean. Under the TSS-based ensemble framework, ROC values of EMmean, EMca, and EMwmean were 0.863 ± 0.011, 0.839 ± 0.014, and 0.863 ± 0.011, respectively, and their TSS values were 0.580 ± 0.023, 0.574 ± 0.022, and 0.581 ± 0.022, respectively. Among the candidate ensemble models, EMwmeanByTSS had the highest TSS value and shared the highest ROC value with EMmean. Considering its acceptable predictive performance and ability to provide continuous suitability outputs for subsequent spatial analyses, EMwmeanByTSS was selected as the final model for suitability projection, environmental variable importance analysis, suitability-area statistics, range-change analysis, and centroid migration analysis (**Supplementary Table 5**).

### 3.2 Environmental variable importance and response curves

**Figure 4:**
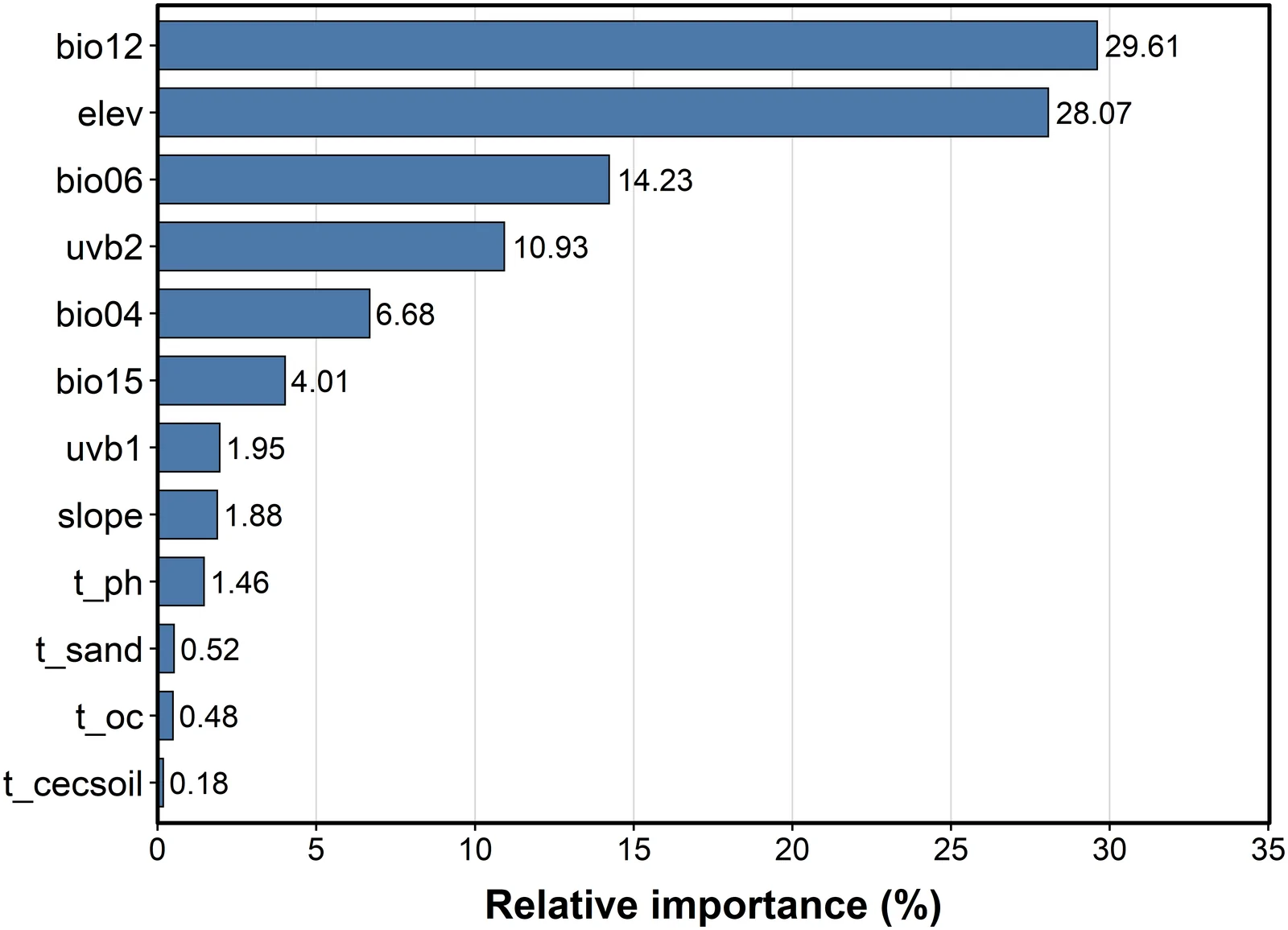
The relative contribution of environmental variables to the potential suitable distribution.

Based on the final EMwmeanByTSS ensemble model, the 12 environmental variables showed clear differences in relative contribution to the potential suitable distribution of *S. italica* (**Table 1**; **Figure 4**). Annual precipitation (bio12) had the highest contribution (29.61%), followed by elevation (elev, 28.07%), minimum temperature of the coldest month (bio06, 14.23%), and UV-B seasonality (uvb2, 10.93%). These four variables together accounted for 82.82% of total model contribution and were therefore the dominant predictors of *S. italica* suitability. Other variables contributed less: temperature seasonality (bio04), precipitation seasonality (bio15), annual mean UV-B radiation (uvb1), and slope contributed 6.68%, 4.01%, 1.95%, and 1.88%, respectively; topsoil pH (t_ph), topsoil sand content (t_sand), topsoil organic carbon (t_oc), and topsoil cation exchange capacity (t_cecsoil) contributed 1.46%, 0.52%, 0.48%, and 0.18%, respectively.

**Figure 5:**
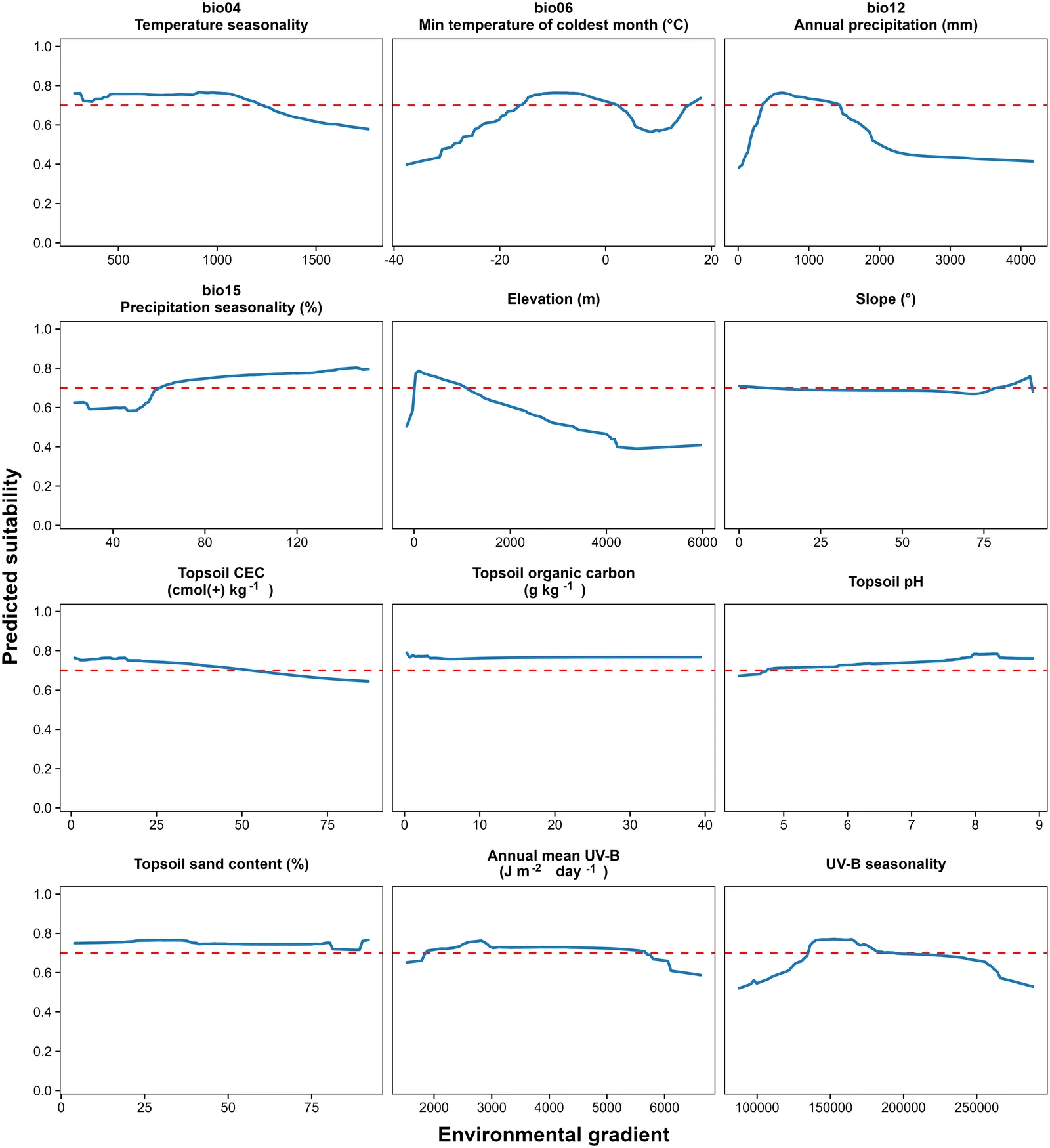
Response curve of environmental variables.

Response curves from the EMwmeanByTSS ensemble showed nonlinear responses of *S. italica* suitability to major environmental gradients (**Figure 5**). Using curve segments near peak suitability or predicted suitability ≥ 0.70 as high-suitability response intervals, *S. italica* maintained relatively high predicted suitability at annual precipitation (bio12) of approximately 500–800 mm, with peak suitability within this range; suitability decreased at higher precipitation levels, dropping to approximately 0.42–0.46 in high-precipitation regions. Along the elevation gradient, predicted suitability was highest in low-elevation areas, peaking at approximately 100–300 m with suitability values of 0.78–0.80; suitability then declined continuously and decreased to approximately 0.40 above 4, 000 m. The response to minimum temperature of the coldest month (bio06) was unimodal, with relatively high predicted suitability at approximately -15 to -5 degrees °C (0.75–0.77), followed by a decline when bio06 increased to 0–10 degrees °C. UV-B seasonality (uvb2) also showed a unimodal response, with relatively high predicted suitability at approximately 1.40–1.70 ×10^5^ and suitability values of 0.76–0.78, followed by a decline under stronger UV-B seasonality.

The response amplitudes of the remaining variables were smaller. Predicted suitability increased with precipitation seasonality (bio15) from approximately 30% to 70%, rising from around 0.60 to about 0.72 and then approaching a plateau of 0.78–0.80 at higher values. Temperature seasonality (bio04) maintained predicted suitability of approximately 0.74–0.76 between 400 and 1, 000 and declined when values exceeded about 1, 200. Annual mean UV-B radiation (uvb1) reached relatively high suitability at approximately 2, 500–3, 000 J m^-2^ day^-1^ and then declined at higher radiation levels. Slope and soil variables showed generally flat responses; topsoil pH showed a slight increasing trend with pH, topsoil organic carbon and sand content varied little across most gradients, and topsoil cation exchange capacity showed a gradual decreasing trend.

### 3.3 Current potential suitable distribution

Under the historical climate baseline, the total potential suitable area of *S. italica* in China was 4.09×10^6^ km^2^, accounting for 44.22% of the study area. Within the suitable area, low-suitability, moderately suitable, and highly suitable areas were 1.84 ×10^6^ km^2^ (19.83%), 1.46 ×10^6^ km^2^ (15.74%), and 0.80 ×10^6^ km^2^ (8.66%), respectively (**Figure 1**; **Table 2**).

**Table 2:** Portions of different classes of potential distribution area of *Setaria Italica* under current and future climate scenarios/years.

Species	Period		Poorly suitable area	Moderately suitable area	Highly suitable area	Extremely highly suitable area	Total suitable area
Area of each suitable area ×10^6^ km^2^ (change in the area compared to current)							
*Setaria Italica*	Current	-	5.16	1.84	1.46	0.80	4.09
	SSP1-2.6	2030	4.93 (-4.42%)	1.89 (+2.96%)	1.58 (+8.32%)	0.85 (+6.59%)	4.32 (+5.58%)
		2050	4.91 (-4.91%)	1.93 (+5.37%)	1.54 (+5.47%)	0.88 (+9.42%)	4.35 (+6.20%)
		2070	4.97 (-3.78%)	1.99 (+8.37%)	1.43 (-1.54%)	0.87 (+8.00%)	4.29 (+4.77%)
		2090	4.86 (-5.85%)	2.05 (+11.46%)	1.43 (-1.72%)	0.92 (+14.57%)	4.40 (+7.38%)
	SSP2-4.5	2030	4.94 (-4.22%)	1.93 (+5.01%)	1.53 (+4.84%)	0.86 (+6.89%)	4.31 (+5.32%)
		2050	4.68 (-9.41%)	2.16 (+17.81%)	1.50 (+2.82%)	0.92 (+14.69%)	4.58 (+11.87%)
		2070	4.60 (-10.83%)	2.20 (+20.09%)	1.50 (+2.67%)	0.95 (+18.91%)	4.65 (+13.66%)
		2090	4.53 (-12.33%)	2.33 (+26.69%)	1.44 (-0.86%)	0.96 (+19.86%)	4.73 (+15.55%)
	SSP3-7.0	2030	4.92 (-4.62%)	1.91 (+4.26%)	1.55 (+6.48%)	0.87 (+8.26%)	4.33 (+5.83%)
		2050	4.78 (-7.45%)	2.11 (+15.21%)	1.51 (+3.39%)	0.86 (+7.02%)	4.48 (+9.40%)
		2070	4.53 (-12.26%)	2.30 (+25.04%)	1.46 (+0.46%)	0.97 (+20.76%)	4.73 (+15.46%)
		2090	4.40 (-14.86%)	2.35 (+28.22%)	1.42 (-2.33%)	1.08 (+35.31%)	4.86 (+18.74%)
	SSP5-8.5	2030	4.93 (-4.53%)	1.96 (+6.62%)	1.51 (+3.83%)	0.86 (+7.09%)	4.33 (+5.72%)
		2050	4.62 (-10.45%)	2.19 (+19.39%)	1.49 (+2.21%)	0.95 (+18.89%)	4.63 (+13.18%)
		2070	4.61 (-10.61%)	2.33 (+26.95%)	1.35 (-7.40%)	0.96 (+20.05%)	4.64 (+13.38%)
		2090	4.39 (-14.96%)	2.36 (+28.75%)	1.45 (-0.30%)	1.05 (+31.07%)	4.87 (+18.87%)

Spatially, highly suitable areas were concentrated mainly in the North China Plain, the eastern Loess Plateau, and the southern margin of Northeast China, including Shanxi, Hebei, Henan, Shandong, northern Shaanxi, southeastern Inner Mongolia, southern Liaoning, and southern Jilin. Moderately suitable areas were distributed around the highly suitable core, including peripheral zones of the Huang-Huai-Hai Plain, southern Northeast China, central China, eastern China, and parts of southwest China. Low-suitability areas were more extensive and occurred in northern Northeast China, central-eastern Inner Mongolia, the middle and lower Yangtze River region, southern hilly regions, and local low-elevation areas of southwest China. Unsuitable areas were mainly distributed in most of Xinjiang, the Qinghai-Tibet Plateau, western Gansu, western Inner Mongolia, and other cold, arid, or high-elevation regions. To further assess the ecological applicability of the suitability threshold (0.190), the predicted suitability values from the final EMwmeanByTSS model were extracted for all occurrence records. Among the 1, 948 occurrence records, 27 records located in NoData areas were excluded, resulting in 1, 921 valid records for threshold evaluation. Of these records, 1, 898 were located within predicted suitable areas (suitability ≥0.190), whereas only 23 records fell below the threshold. The omission rate was therefore 1.20%, indicating that the selected threshold captured 98.80% of known occurrence records and provided a reasonable basis for distinguishing suitable and unsuitable areas in subsequent spatial analyses of *S. italica* in China.

### 3.4 Future changes in suitability classes

**Figure 6:**
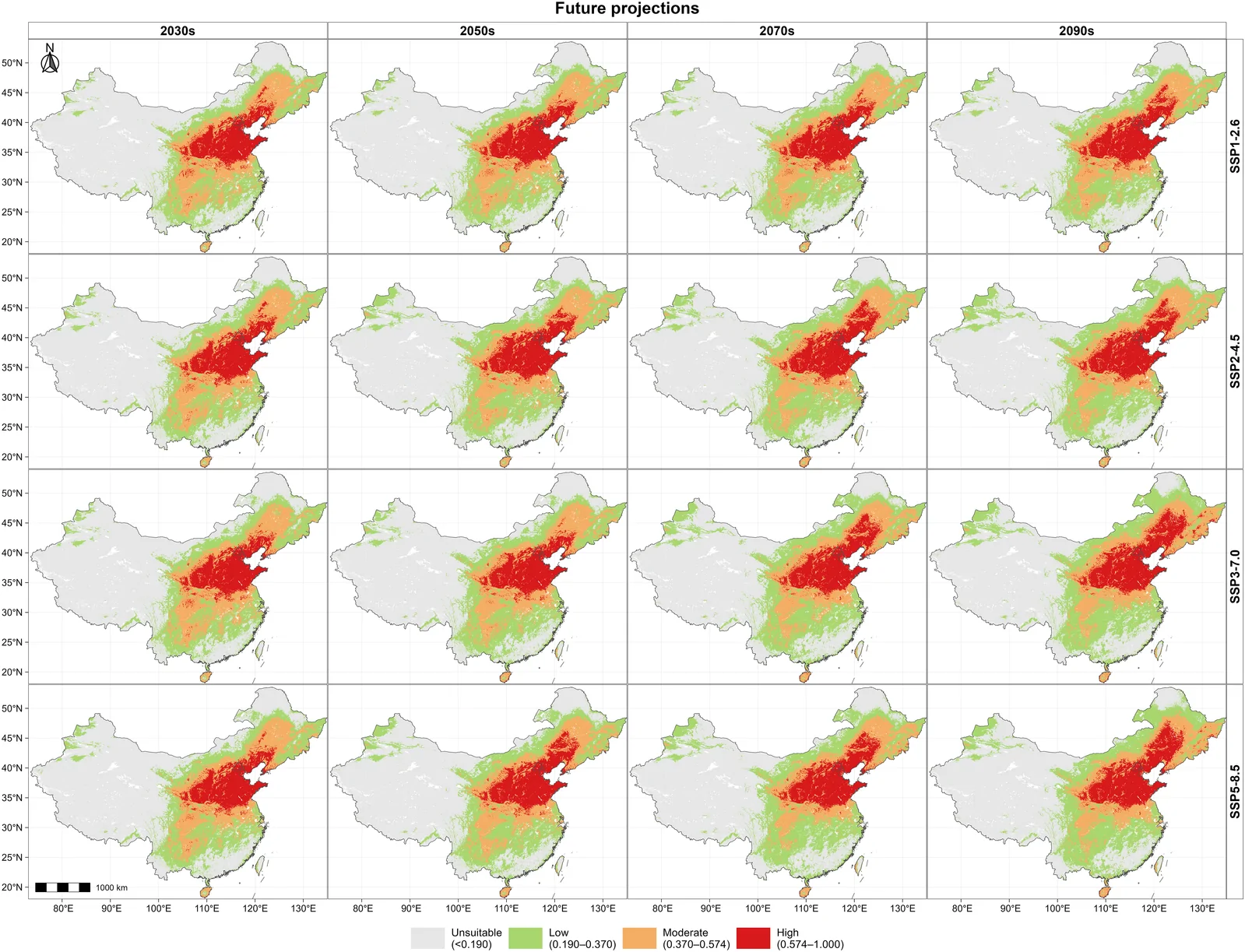
The potential distribution range of *Setaria Italica* under future climate change scenarios.

Under future SSP scenarios, total suitable area ranged from 4.29 ×10^6^ km^2^ to 4.87 ×10^6^ km^2^, representing an increase of 4.77%–18.87% relative to the baseline. Total suitable area was 4.31 ×10^6^ –4.33 ×10^6^ km^2^ in the 2030s, 4.35 ×10^6^ – 4.63 ×10^6^ km^2^ in the 2050s, 4.29 ×10^6^ – 4.73 ×10^6^ km^2^ in the 2070s, and 4.40 ×10^6^ – 4.87 ×10^6^ km^2^ in the 2090s. The largest total suitable area occurred under SSP5-8.5 in the 2090s (4.87 ×10^6^ km^2^), followed by SSP3-7.0 in the 2090s (4.86×10^6^ km^2^) (**Figure 6**; **Table 2**). Low-suitability area changed from 1.84 ×10^6^ km^2^ under the baseline to 1.89 ×10^6^ –2.36 ×10^6^ km^2^ under future scenarios, corresponding to changes of 2.96%–28.75%, with the maximum under SSP5-8.5 in the 2090s. Highly suitable area increased from 0.80 ×10^6^ km^2^ under the baseline to 0.85 ×10^6^ –1.08 ×10^6^ km^2^, corresponding to changes of 6.59%–35.31%; the largest highly suitable area occurred under SSP3-7.0 in the 2090s (1.08 ×10^6^ km^2^). Moderately suitable area changed from 1.46 ×10^6^ km^2^ under the baseline to 1.35 ×10^6^ –1.58 ×10^6^ km^2^. Future highly suitable areas remained concentrated in the North China Plain, the eastern Loess Plateau, and the southern margin of Northeast China. In the 2070s-2090s, under SSP3-7.0 and SSP5-8.5, the continuity of highly suitable areas increased in southern Northeast China, southeastern Inner Mongolia, and the northern margin of North China. Low-suitability and moderately suitable areas expanded mainly around the periphery of the highly suitable core, particularly toward Northeast China, central-eastern Inner Mongolia, central China, and local parts of southwest China.

### 3.5 Range expansion and contraction

Overlay analysis of baseline and future binary suitable areas identified stable suitable, stable unsuitable, expansion, and contraction zones. Stable suitable areas were mainly distributed in North China, the eastern Loess Plateau, the southern margin of Northeast China, the Huang-Huai-Hai Plain, and parts of central-eastern China. Stable unsuitable areas were concentrated in Xinjiang, the Qinghai-Tibet Plateau, western Gansu, western Inner Mongolia, and other cold or extremely arid regions.

**Figure 7:**
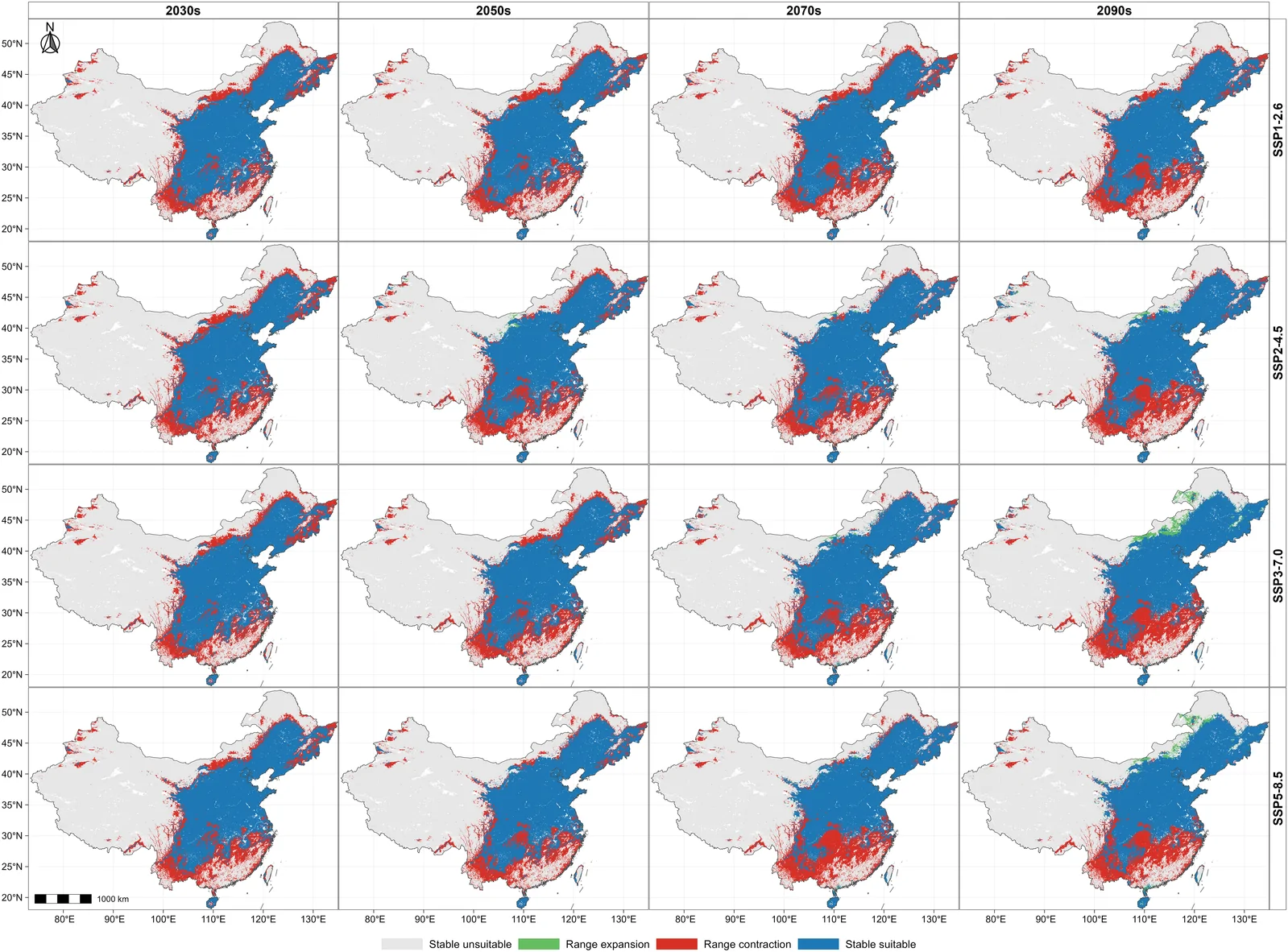
The potential distribution range changes of *Setaria Italica* under future climate change scenarios.

Expansion exceeded contraction under all future periods and scenarios. In the 2030s, expansion area was 0.26 ×10^6^ –0.34 ×10^6^ km^2^, contraction area was 0.03 ×10^6^–0.10 ×10^6^ km^2^, and net gain was 0.22 ×10^6^ – 0.24 ×10^6^ km^2^. In the 2050s, expansion, contraction, and net gain were 0.31 ×10^6^ – 0.63 ×10^6^ km^2^, 0.06 ×10^6^ –0.11 ×10^6^ km^2^, and 0.25 ×10^6^–0.54 ×10^6^ km^2^, respectively. In the 2070s, they were 0.31 ×10^6^–0.80 ×10^6^ km^2^, 0.10 ×10^6^–0.24 ×10^6^ km^2^, and 0.20 ×10^6^-0.63 ×10^6^ km^2^, respectively. In the 2090s, expansion area increased to 0.44 ×10^6^–0.98 ×10^6^ km^2^, contraction area was 0.14 ×10^6^–0.20 ×10^6^ km^2^, and net gain was 0.30 ×10^6^–0.77 ×10^6^ km^2^. The largest net gains occurred under SSP3-7.0 and SSP5-8.5 in the 2090s (0.77 ×10^6^ km^2^) (**Figure 7**; **Table 2**). Spatially, expansion zones occurred mainly along the periphery and northern margins of the current suitable region, including northern Northeast China, central-eastern Inner Mongolia, the margins of the Loess Plateau, and several transitional regions in central and western China. Contraction zones were mainly scattered along the southern and southwestern margins of the current suitable region and several mid- to low-latitude transitional areas.

### 3.6 Centroid migration

**Figure 8:**
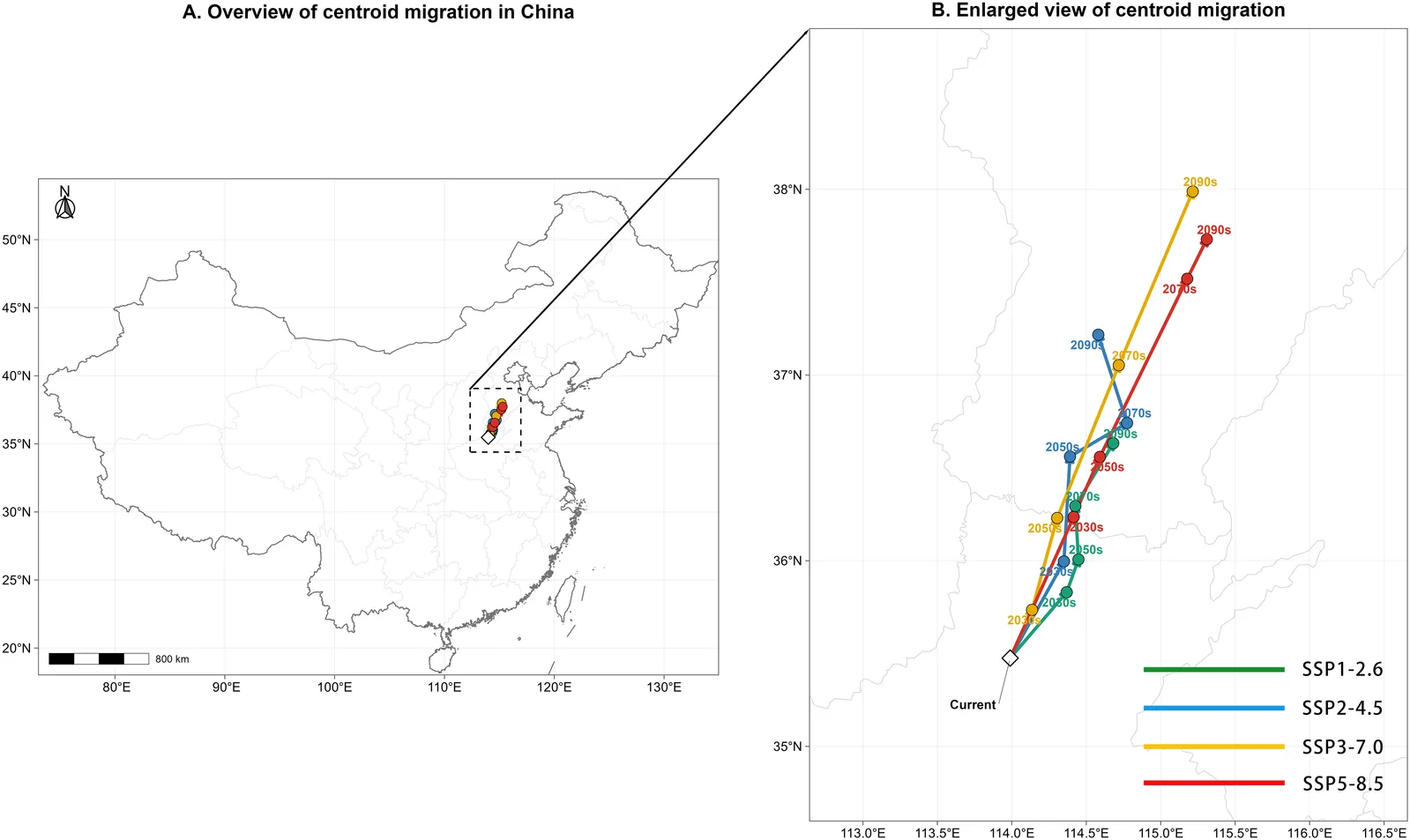
The migration routes of *Setaria Italica* under future climate change scenarios.

Under the historical baseline, the centroid of the potential suitable area was located at 35.32°N, 113.47°E, in northern Henan Province, near the transition zone between the southern Taihang Mountains and the North China Plain. Under future SSP scenarios, the centroid generally shifted from northern Henan toward southern–central Shanxi, with an overall northward to northwestward direction. By the 2090s, the centroid shifted to 36.30°N, 113.11°E under SSP1-2.6, with a net displacement of 113.33 km; to 36.88°N, 112.16°E under SSP2-4.5, with a net displacement of 209.14 km; to 37.29°N, 112.59°E under SSP3-7.0, with the largest net displacement of 232.79 km; and to 37.08°N, 112.79°E under SSP5-8.5, with a net displacement of 204.05 km. Across future periods, centroid displacement increased from 29.98–94.50 km in the 2030s to 49.70–157.90 km in the 2050s, 82.74–192.66 km in the 2070s, and 113.33–232.79 km in the 2090s. Overall, the centroid migration indicated a moderate northward–northwestward shift from northern Henan toward southern–central Shanxi, with increasing displacement under intensified emission scenarios. Despite reaching 232.79 km under SSP3-7.0 by the 2090s, the centroid remained within the northern dryland agricultural region of China, suggesting a directional adjustment of suitable habitats rather than a substantial geographical expansion (**Figure 8**; **Supplementary Table 6**).

## 4 Discussion

### 4.1 Ensemble modelling improved stability of suitability prediction

Using 1, 948 occurrence records and multiple environmental predictors, this study applied eight single algorithms and ensemble modelling to predict the potential suitable distribution of *S. italica* in China. Predictive performance differed among single algorithms, with RF, MAXENT, MARS, and XGBOOST generally outperforming CTA and FDA. Such algorithmic differences are common in SDMs because models differ in their ability to represent nonlinear responses, interactions, and threshold-like relationships. Ensemble forecasting has been widely recommended to reduce dependence on any single algorithm and to improve the stability and interpretability of spatial projections (Araujo and New, 2007a; Elith and Leathwick, 2009; Thuiller et al., 2009).

The final EMwmeanByTSS model achieved validation ROC and TSS values of 0.863 ± 0.011 and 0.581 ± 0.022, respectively, indicating good discrimination ability and acceptable overall predictive performance. In addition, the ensemble framework reduced uncertainties associated with individual algorithms and provided more stable predictions under different climatic scenarios. However, some uncertainty may remain because Chinese foxtail millet comprises spring-sown and summer-sown ecotypes with distinct phenological characteristics and hydrothermal requirements (Jia et al., 2020). Therefore, the model outputs should be interpreted as representing the overall climatic suitability of *S. italica* at the national scale rather than the specific niche of a single ecotype. This interpretation is consistent with the principle that SDM outputs should be evaluated according to data characteristics, model assumptions, and the intended application purpose (Guillera-Arroita et al., 2015).

### 4.2 Water availability, elevation, and cold limitation jointly structured high suitability

Annual precipitation, elevation, minimum temperature of the coldest month, and UV-B seasonality together explained 82.82% of model contribution, indicating that *S. italica* suitability in China is structured primarily by moisture conditions, topographic-thermal gradients, cold limitation, and radiation seasonality (Zhang et al., 2012; Lata et al., 2013). Annual precipitation was the strongest predictor, with high predicted suitability around 500–800 mm. This range is consistent with the dryland-crop nature of foxtail millet: the crop is drought tolerant and water efficient, but high suitability does not correspond to extreme aridity (Bennetzen et al., 2012; Yang et al., 2024). Instead, its advantage lies in maintaining growth and yield under limited but not severely deficient water supply.

The decline in suitability under excessive precipitation may reflect the agricultural-ecological context of the crop (Challinor et al., 2014). Humid regions of southern China may provide abundant water, but high humidity, disease risk, rainfall during maturation, and competition from rice- and maize-based cropping systems may reduce the relative suitability of *S. italica*. In contrast, the North China Plain, eastern Loess Plateau, and southern margin of Northeast China combine moderate precipitation, marked seasonality, and sufficient heat resources (Rosenzweig et al., 2014). More generally, climate impacts on crops are regionally heterogeneous, and suitability changes should not be interpreted as simple expansion or contraction under warming (Lobell et al., 2011).

Elevation was the second most important predictor, with highest suitability at low elevations and a sharp decline at high elevations (Lata et al., 2013). Elevation is not merely a topographic variable; it integrates temperature regime, growing-season length, radiation, soil development, and agricultural accessibility (Lata et al., 2013). Although *S. italica* has a short growth period and strong stress tolerance, high-elevation regions are often associated with low temperatures, short growing seasons, and complex terrain, all of which can constrain continuous suitability (Huang et al., 2023). Thus, elevation should be interpreted as a compound topographic-climatic constraint rather than as a single geomorphic factor.

The importance of minimum temperature of the coldest month is also ecologically meaningful. Although *S. italica* is a warm-season annual crop and does not overwinter, this variable captures broad climate-zone differences, frost limitation, growing-season constraints, and farming-system background (Zhang et al., 2012). Experimental work has shown that ambient temperature affects heading date in *S. italica* and that lower temperatures can alter developmental timing among accessions (Huang et al., 2023). In this study, high suitability occurred under relatively cold but not extremely cold conditions, indicating that the most suitable regions are temperate dryland agricultural zones rather than alpine or humid tropical zones.

UV-B seasonality also contributed substantially to the model, suggesting that radiation regimes participate in shaping the potential distribution of *S. italica*. Unlike many C3 crops, foxtail millet is a C4 crop characterized by high photosynthetic efficiency, high light saturation capacity, and strong resource-use efficiency, which may confer adaptive advantages in environments with strong solar radiation and seasonal drought stress (Li and Brutnell, 2011; Bennetzen et al., 2012). Moderate UV-B exposure can activate UV-B perception pathways, particularly UVR8-mediated signaling, and stimulate the accumulation of protective metabolites such as flavonoids and phenolic compounds, thereby enhancing antioxidant capacity and photoprotection (Jansen et al., 1998; Jenkins, 2009). These responses may improve the ability of S. italica to maintain photosynthetic performance under high-radiation environments. However, excessive UV-B exposure can induce oxidative stress, damage photosynthetic systems, and reduce plant productivity (Jansen et al., 1998; Ballaré et al., 2011). Therefore, the unimodal response observed in this study suggests that UV-B effects are not simply positive, but instead reflect a balance between radiation-driven acclimation and physiological stress. At the national scale, UV-B seasonality may also integrate broader geographic gradients associated with latitude, elevation, temperature, and moisture availability, thereby contributing to the spatial differentiation of suitable habitats. Soil variables had comparatively low contribution at the national scale, suggesting that they are more likely to influence field-level performance than broad distributional boundaries, especially because agronomic management can partly modify soil constraints (Zhao et al., 2021). In addition, the relatively coarse spatial resolution of the HWSD soil dataset (~5 km) may have limited its ability to capture fine-scale soil heterogeneity associated with micro-topography, field management practices, and local variation. Therefore, the contribution of soil variables may have been underestimated in this macro-scale modelling framework. Future studies integrating higher-resolution soil datasets and field-based soil measurements may provide further insights into the role of soil conditions in shaping the distribution of *S. italica*.

### 4.3 Current highly suitable areas correspond to the historical core of northern millet agriculture

Under the current climate, highly suitable areas were concentrated in the North China Plain, the eastern Loess Plateau, and the southern margin of Northeast China. This spatial pattern corresponds closely to the historical core of millet agriculture in northern China. Archaeological evidence indicates that millets played a central role in the emergence and spread of early dryland agriculture across the Yellow River Basin and northern China (Crawford, 2009; Lu et al., 2009). The agreement between modelled high suitability and long-term cultivation regions suggests that the predicted suitable area captures not only contemporary environmental gradients but also the broad ecological context within which *S. italica* domestication and cultivation developed (Hunt et al., 2008).

This pattern is also consistent with the geographic differentiation of *S. italica* germplasm. Previous studies have shown pronounced population structure, genetic diversity, and regional adaptation among Chinese *S. italica* landraces (Wang et al., 2010; Jia et al., 2013). The concentration of highly suitable areas in Shanxi, Hebei, Henan, Shandong, northern Shaanxi, southeastern Inner Mongolia, Liaoning, and Jilin overlaps with major traditional cultivation and germplasm regions. Thus, the current suitability pattern likely reflects the joint influence of water availability, temperature seasonality, low-elevation landscapes, historical cultivation, and human selection.

Low- and moderate-suitability areas formed gradients around the highly suitable core and extended toward Northeast China, central China, eastern China, and parts of southwest China. Northern Northeast China may remain limited by cold stress and growing-season length, while humid southern regions may be constrained by excessive rainfall and contrasting cropping systems. Southwestern areas are further influenced by fragmented terrain and elevation gradients. Therefore, current suitability is not a simple latitudinal pattern but a composite outcome of climate zones, landforms, and agricultural ecological regions.

### 4.4 Future expansion reflected marginal improvement rather than a wholesale shift of the core

Under future SSP scenarios, total suitable area generally increased, especially under SSP3-7.0 and SSP5-8.5 in the 2090s. This pattern is consistent with the possibility that warming may alleviate cold limitations in some northern or transitional regions. The CMIP6 ScenarioMIP and SSP/RCP frameworks provide a basis for comparing climate-change risks across alternative socioeconomic and emission pathways (Eyring et al., 2016; O’Neill et al., 2016). For *S. italica*, warming may increase potential suitability along the margins of Northeast China, central-eastern Inner Mongolia, and the Loess Plateau, where cold limitation currently constrains suitability.

Importantly, future expansion occurred mainly around the periphery and northern margins of the existing suitable region, while highly suitable core areas remained anchored in the North China Plain, eastern Loess Plateau, and southern Northeast China. This indicates a pattern of stable core suitability, marginal expansion, and local contraction rather than a wholesale relocation of the suitability core (Rötter et al., 2011). Expansion should therefore not be interpreted as immediate agricultural feasibility. Potential environmental suitability must be considered together with land availability, water resources, cropping systems, mechanization, and varietal adaptation (Rötter et al., 2011; Rosenzweig et al., 2014; Jägermeyr et al., 2021).

Suitability-class changes further showed that low- and high-suitability areas tended to increase, whereas moderate-suitability areas fluctuated among scenarios and periods. This indicates that future change involves class redistribution rather than uniform expansion of all classes. Some currently unsuitable regions may first become low-suitability areas, whereas some moderate-suitability regions may become highly suitable as water-thermal conditions improve. Conversely, some transitional areas may lose suitability because of humid-heat stress or seasonal mismatch. This nonlinear reorganization is consistent with the response curves, which showed unimodal or threshold-like responses to precipitation, elevation, temperature, and UV-B seasonality (Zhao et al., 2021).

### 4.5 Local contraction indicates risk along southern and transitional margins

Although the total suitable area increased under future scenarios, contraction zones persisted, mainly along the southern and southwestern margins of the current suitable region and in several mid- to low-latitude transitional areas. These results indicate that climate change does not simply expand suitability for foxtail millet. Southern and southwestern margins may face increasing heat, rainfall, humidity, disease risk, or seasonal mismatch, causing local declines in potential suitability. A climate-smart management study in Lishu County, Jilin Province, also showed that future climate change can produce scenario- and period-dependent effects on *S. italica* production and that adaptive management is required to reduce risks (Liu et al., 2020).

The contraction pattern is consistent with the response curve showing reduced suitability under high annual precipitation. Drought tolerance does not imply adaptation to humid conditions. Excessive rainfall and humidity may increase lodging, disease pressure, and adverse weather during maturation and may reduce the competitiveness of *S. italica* within local cropping systems. Therefore, contraction zones should not be ignored in planning. These areas warrant climate-risk monitoring, variety screening for heat, humidity, disease, and lodging tolerance, and adjustment of sowing dates and cultivation practices.

### 4.6 Centroid displacement indicated limited spatial reorganization

Centroid analysis showed that the historical suitability centroid was located near the Jiaozuo-Xinxiang border in northern Henan, while future centroids shifted toward southern and central Shanxi by the 2090s. Net displacement ranged from 113.33 to 232.79 km, mainly northward to northwestward, with the largest displacement under SSP3-7.0 in the 2090s. This shift is consistent with enhanced suitability along northern margins and increased continuity of suitable areas in southern Northeast China and southeastern Inner Mongolia.

Nevertheless, centroid displacement remained limited, and centroids in all future scenarios stayed within the northern dryland agricultural region of China. Thus, future redistribution should be interpreted as marginal expansion and spatial rebalancing around an existing core rather than long-distance relocation. The centroid results support the area-change analysis: stable suitable areas remain dominant, expansion occurs mainly along the margins, and contraction remains local. Accordingly, the northward/northwestward centroid movement signals enhanced suitability in northern marginal and Shanxi transitional zones but does not imply a decline in the importance of existing core regions.

### 4.7 Implications for production planning and resource management

The results provide spatial guidance for *S. italica* production planning and climate-adaptive management in China. Existing highly suitable core regions, mainly located in the North China Plain, the eastern Loess Plateau, and the southern margin of Northeast China, should remain priorities for stabilizing production, improving quality, conserving germplasm, and promoting water-saving and stress-resilient cultivation. These regions cover parts of Shanxi, Hebei, Henan, Shandong, northern Shaanxi, southeastern Inner Mongolia, southern Liaoning, and southern Jilin. Because annual precipitation, elevation, cold limitation, and UV-B seasonality were the dominant predictors, management in these areas should focus on precipitation variability, drought episodes, and extreme climate events (Jägermeyr et al., 2021). More broadly, climate-resilient agriculture requires integrated variety improvement, agronomic management, ecological regulation, and risk monitoring (Challinor et al., 2014; Altieri et al., 2015). In addition, future UV-B radiation projections consistent with CMIP6 scenarios are currently unavailable; therefore, UV-B variables were kept unchanged during future simulations. Although UV-B radiation was identified as an important predictor of *S. italica* suitability, potential future changes associated with atmospheric composition and ozone dynamics were not explicitly considered. Future integration of dynamic UV-B projections with climate scenarios may further improve the assessment of crop suitability under climate change.

Potential expansion regions should be interpreted cautiously. Northern Northeast China, central-eastern Inner Mongolia, Loess Plateau margins, and central Shanxi may become more environmentally suitable under future scenarios, but whether these regions can support stable production depends on cultivated land availability, water-resource carrying capacity, mechanization, varietal adaptation, and local agricultural infrastructure (Liu et al., 2020). These regions are therefore better considered priorities for regional field trials and variety-adaptation evaluation rather than immediate large-scale expansion. Similar climate-adaptive crop studies have emphasized that projected suitability or yield potential should be validated through regional trials and adaptive agronomic management before practical deployment (Challinor et al., 2014; Rosenzweig et al., 2014).

Conversely, contraction-prone areas along the southern, southwestern, and transitional margins require targeted risk management. Although total suitable area increased under future scenarios, local suitability declines may still occur where excessive precipitation, humid–hot conditions, altered seasonal climate patterns, and cropping-system competition reduce environmental suitability (Liu et al., 2020). In these areas, management should prioritize heat- and disease-resistant cultivars, sowing-date adjustment, drainage improvement, and monitoring of climate-related production risks (Lobell et al., 2011; Jägermeyr et al., 2021).

Importantly, for cultivated crops, SDM outputs should be interpreted as broad-scale potential environmental suitability rather than actual planting boundaries. Although environmental conditions determine the fundamental suitability of *S. italica*, realized cultivation patterns are additionally shaped by land use, irrigation availability, socioeconomic conditions, market demand, varietal selection, and local farming traditions (Challinor et al., 2014; Rosenzweig et al., 2014). Consequently, the omission of land-use constraints may result in an overestimation of areas that are environmentally suitable but not realistically available for millet cultivation. For example, some predicted suitable regions may correspond to forests, grasslands, urban areas, ecological conservation zones, or other non-cultivated land types. Under China’s current agricultural conditions, where cultivated land protection and ecological conservation policies strongly regulate land allocation, environmental suitability alone cannot fully represent feasible planting potential (Guillera-Arroita et al., 2015; Zabel et al., 2019). Future studies integrating high-resolution land-use datasets, irrigation information, and agricultural management factors could further distinguish environmentally suitable areas from realistically cultivable regions. Therefore, the predicted suitable areas should support, rather than replace, regional agronomic decision-making. In addition, all occurrence records were analyzed jointly without separating spring-sown and summer-sown ecotypes. Although this integrated approach is appropriate for evaluating national-scale climatic suitability, it may reduce the ability to capture ecotype-specific responses, particularly in transitional regions where different cultivation systems overlap. Future studies incorporating ecotype-level occurrence records, sowing dates, and accumulated temperature zones could further improve regional suitability assessments.

## 5 Conclusions

Using 1, 948 occurrence records and multi-source environmental predictors, this study applied biomod2 ensemble species distribution modelling to evaluate the current and future potential suitable distribution of *S. italica* in China. The selected EMwmeanByTSS ensemble showed good discrimination ability and acceptable predictive performance, supporting its use for national-scale suitability assessment. Environmental variable analysis indicated that annual precipitation, elevation, minimum temperature of the coldest month, and UV-B seasonality were the dominant predictors, jointly contributing 82.82% to the model. These results suggest that the potential suitability of *S. italica* is primarily constrained by water availability, topographic–thermal gradients, cold limitation, and radiation seasonality.

Under the historical baseline, the potential suitable area of *S. italica* covered 4.09 × 10^6^ km², accounting for 44.22% of China’s land area, with highly suitable areas mainly distributed in the North China Plain, the eastern Loess Plateau, and the southern margin of Northeast China. Under future BCC-CSM2-MR CMIP6 projections, total suitable area increased to 4.29 × 10^6^ –4.87 × 10^6^ km² across SSP1-2.6, SSP2-4.5, SSP3-7.0, and SSP5-8.5, indicating a general expansion of potential suitability under future climate change. However, this expansion was spatially uneven, with stable suitability in the current core region, expansion along northern and peripheral margins, and localized contraction along southern, southwestern, and transitional margins.

Overall, future climate change is projected to drive a spatial reorganization rather than a wholesale displacement of *S. italica* suitability in China. The suitability centroid shifted only moderately from northern Henan toward southern–central Shanxi, with a maximum net displacement of 232.79 km, indicating limited northward–northwestward movement within the northern dryland agricultural region. These findings highlight the continued importance of existing core production regions for stable production and germplasm conservation, while projected expansion zones should be prioritized for regional variety trials and adaptive field validation rather than immediate large-scale cultivation.

## Data Availability

The original contributions presented in the study are included in the article/**Supplementary Material**. Further inquiries can be directed to the corresponding author.
