# Supplementary material for: Climate-driven redistribution of foxtail millet (Setaria italica) suitability in China revealed by biomod2 ensemble models: implications for dryland crop adaptation
Source: Front Plant Sci. 2026 Sep 9;17:1882902. doi: 10.3389/fpls.2026.1882902 (PMC13597327; doi:10.3389/fpls.2026.1882902)
Supplement: Supplementary file 1 [file Table1.docx]

**Table S1.** Geographic coordinates of *Setaria Italica* occurrence records used for modelling

| **No.** | **Longitude (°E)** | **Latitude (°N)** | **No.** | **Longitude (°E)** | **Latitude (°N)** | **No.** | **Longitude (°E)** | **Latitude (°N)** | **No.** | **Longitude (°E)** | **Latitude (°N)** |
| --- | --- | --- | --- | --- | --- | --- | --- | --- | --- | --- | --- |
| 1 | 126.183333 | 48.516667 | 2 | 127.450000 | 50.250000 | 3 | 127.333333 | 49.433333 | 4 | 126.550000 | 45.716667 |
| 5 | 127.483333 | 50.250000 | 6 | 128.466667 | 49.583333 | 7 | 125.216667 | 49.166667 | 8 | 126.966667 | 47.433333 |
| 9 | 126.100000 | 47.600000 | 10 | 125.883333 | 48.050000 | 11 | 126.500000 | 48.233333 | 12 | 127.483333 | 46.883333 |
| 13 | 128.016667 | 46.983333 | 14 | 124.850000 | 48.483333 | 15 | 126.250000 | 48.033333 | 16 | 125.300000 | 47.900000 |
| 17 | 127.100000 | 47.233333 | 18 | 124.483333 | 47.800000 | 19 | 126.983333 | 46.633333 | 20 | 111.666667 | 40.833333 |
| 21 | 124.833333 | 47.183333 | 22 | 123.183333 | 47.333333 | 23 | 123.950000 | 47.333333 | 24 | 123.500000 | 47.916667 |
| 25 | 109.750000 | 38.300000 | 26 | 124.250000 | 46.466667 | 27 | 123.416667 | 46.400000 | 28 | 129.500000 | 42.883333 |
| 29 | 112.700000 | 38.416667 | 30 | 125.400000 | 46.500000 | 31 | 125.316667 | 46.066667 | 32 | 125.250000 | 45.700000 |
| 33 | 125.083333 | 45.500000 | 34 | 126.100000 | 46.683333 | 35 | 126.266667 | 46.250000 | 36 | 125.900000 | 47.200000 |
| 37 | 117.033333 | 30.533333 | 38 | 126.133333 | 46.683333 | 39 | 130.566667 | 46.250000 | 40 | 129.900000 | 46.733333 |
| 41 | 129.566667 | 46.333333 | 42 | 105.833333 | 28.800000 | 43 | 130.566667 | 45.750000 | 44 | 132.200000 | 46.316667 |
| 45 | 131.983333 | 47.250000 | 46 | 130.716667 | 47.016667 | 47 | 130.300000 | 46.800000 | 48 | 130.816667 | 47.566667 |
| 49 | 130.116667 | 44.050000 | 50 | 130.900000 | 45.333333 | 51 | 127.966667 | 45.216667 | 52 | 129.383333 | 44.566667 |
| 53 | 129.483333 | 44.366667 | 54 | 128.316667 | 45.450000 | 55 | 131.883333 | 45.550000 | 56 | 129.600000 | 44.566667 |
| 57 | 130.550000 | 44.933333 | 58 | 128.883333 | 47.716667 | 59 | 126.966667 | 45.533333 | 60 | 127.483333 | 45.750000 |
| 61 | 128.033333 | 45.950000 | 62 | 126.300000 | 45.483333 | 63 | 126.600000 | 46.000000 | 64 | 127.416667 | 46.116667 |
| 65 | 126.483333 | 46.866667 | 66 | 128.733333 | 45.966667 | 67 | 126.766667 | 45.750000 | 68 | 127.150000 | 44.916667 |
| 69 | 126.500000 | 44.750000 | 70 | 124.983333 | 42.916667 | 71 | 125.650000 | 43.516667 | 72 | 125.716667 | 44.533333 |
| 73 | 124.416667 | 43.083333 | 74 | 124.316667 | 43.300000 | 75 | 124.800000 | 43.516667 | 76 | 125.283333 | 43.333333 |
| 77 | 125.033333 | 43.433333 | 78 | 122.833333 | 45.633333 | 79 | 123.083333 | 44.750000 | 80 | 124.266667 | 45.500000 |
| 81 | 124.816667 | 45.166667 | 82 | 125.166667 | 44.433333 | 83 | 123.116667 | 44.500000 | 84 | 127.333333 | 43.716667 |
| 85 | 125.866667 | 41.733333 | 86 | 126.516667 | 43.700000 | 87 | 123.066667 | 44.783333 | 88 | 126.750000 | 42.966667 |
| 89 | 124.016667 | 45.000000 | 90 | 123.500000 | 43.516667 | 91 | 128.183333 | 41.416667 | 92 | 124.816667 | 45.116667 |
| 93 | 126.950000 | 44.416667 | 94 | 125.833333 | 44.150000 | 95 | 128.200000 | 43.366667 | 96 | 121.466667 | 45.050000 |
| 97 | 121.250000 | 43.583333 | 98 | 121.750000 | 42.716667 | 99 | 126.466667 | 43.950000 | 100 | 120.666667 | 42.850000 |
| 101 | 122.250000 | 43.633333 | 102 | 121.300000 | 45.050000 | 103 | 122.333333 | 42.950000 | 104 | 120.866667 | 44.550000 |
| 105 | 125.516667 | 42.666667 | 106 | 125.900000 | 41.683333 | 107 | 126.816667 | 42.350000 | 108 | 125.733333 | 42.283333 |
| 109 | 122.500000 | 45.400000 | 110 | 126.050000 | 42.950000 | 111 | 126.150000 | 41.100000 | 112 | 127.266667 | 42.416667 |
| 113 | 129.750000 | 43.316667 | 114 | 123.966667 | 44.250000 | 115 | 123.250000 | 44.333333 | 116 | 118.966667 | 42.266667 |
| 117 | 126.033333 | 42.666667 | 118 | 123.450000 | 41.733333 | 119 | 124.033333 | 42.550000 | 120 | 122.816667 | 41.983333 |
| 121 | 124.900000 | 42.100000 | 122 | 125.033333 | 41.733333 | 123 | 123.400000 | 42.500000 | 124 | 123.783333 | 41.316667 |
| 125 | 124.100000 | 42.783333 | 126 | 123.350000 | 42.750000 | 127 | 123.933333 | 41.883333 | 128 | 123.866667 | 42.300000 |
| 129 | 122.716667 | 41.516667 | 130 | 125.350000 | 41.250000 | 131 | 122.600000 | 40.200000 | 132 | 118.650000 | 43.533333 |
| 133 | 118.950000 | 42.250000 | 134 | 118.683333 | 41.916667 | 135 | 119.033333 | 42.950000 | 136 | 120.450000 | 41.550000 |
| 137 | 123.483333 | 48.116667 | 138 | 119.883333 | 42.283333 | 139 | 119.333333 | 41.550000 | 140 | 118.033333 | 43.600000 |
| 141 | 119.783333 | 40.800000 | 142 | 119.633333 | 41.383333 | 143 | 119.383333 | 41.233333 | 144 | 119.716667 | 41.083333 |
| 145 | 120.750000 | 41.816667 | 146 | 121.650000 | 42.033333 | 147 | 120.350000 | 40.350000 | 148 | 120.716667 | 40.616667 |
| 149 | 121.233333 | 41.533333 | 150 | 122.016667 | 41.683333 | 151 | 121.366667 | 41.316667 | 152 | 122.550000 | 42.400000 |
| 153 | 120.850000 | 40.750000 | 154 | 121.783333 | 41.600000 | 155 | 121.116667 | 41.133333 | 156 | 122.350000 | 40.416667 |
| 157 | 122.716667 | 40.883333 | 158 | 123.166667 | 41.233333 | 159 | 123.283333 | 40.283333 | 160 | 122.383333 | 41.383333 |
| 161 | 122.966667 | 39.700000 | 162 | 124.033333 | 40.450000 | 163 | 124.766667 | 40.716667 | 164 | 124.333333 | 40.050000 |
| 165 | 121.233333 | 38.816667 | 166 | 121.950000 | 39.400000 | 167 | 122.016667 | 41.183333 | 168 | 122.266667 | 40.666667 |
| 169 | 122.566667 | 39.266667 | 170 | 121.700000 | 39.083333 | 171 | 123.000000 | 41.083333 | 172 | 113.150000 | 41.033333 |
| 173 | 113.666667 | 40.416667 | 174 | 113.166667 | 40.450000 | 175 | 112.500000 | 40.533333 | 176 | 113.866667 | 40.866667 |
| 177 | 111.683333 | 41.516667 | 178 | 111.416667 | 41.116667 | 179 | 111.800000 | 40.416667 | 180 | 110.533333 | 40.550000 |
| 181 | 110.016667 | 40.400000 | 182 | 109.983333 | 39.816667 | 183 | 108.833333 | 38.600000 | 184 | 111.683333 | 39.966667 |
| 185 | 111.183333 | 40.266667 | 186 | 108.633333 | 40.766667 | 187 | 110.016667 | 40.616667 | 188 | 107.233333 | 40.766667 |
| 189 | 106.133333 | 38.333333 | 190 | 106.700000 | 38.800000 | 191 | 107.400000 | 37.783333 | 192 | 105.183333 | 37.516667 |
| 193 | 105.666667 | 37.483333 | 194 | 105.083333 | 35.683333 | 195 | 107.183333 | 35.683333 | 196 | 108.016667 | 35.816667 |
| 197 | 107.983333 | 36.466667 | 198 | 107.300000 | 36.566667 | 199 | 107.900000 | 35.983333 | 200 | 107.400000 | 35.150000 |
| 201 | 107.350000 | 35.333333 | 202 | 106.650000 | 35.533333 | 203 | 108.000000 | 35.416667 | 204 | 103.816667 | 36.050000 |
| 205 | 107.016667 | 35.283333 | 206 | 108.366667 | 35.483333 | 207 | 106.633333 | 35.216667 | 208 | 105.333333 | 34.733333 |
| 209 | 104.883333 | 34.733333 | 210 | 105.716667 | 34.583333 | 211 | 105.666667 | 34.866667 | 212 | 104.616667 | 35.583333 |
| 213 | 103.933333 | 36.366667 | 214 | 105.250000 | 35.200000 | 215 | 100.466667 | 38.933333 | 216 | 103.350000 | 36.083333 |
| 217 | 116.516667 | 28.266667 | 218 | 104.633333 | 35.000000 | 219 | 103.250000 | 36.750000 | 220 | 102.683333 | 37.916667 |
| 221 | 101.966667 | 38.233333 | 222 | 105.716667 | 33.750000 | 223 | 104.683333 | 36.566667 | 224 | 104.050000 | 37.183333 |
| 225 | 104.116667 | 35.833333 | 226 | 105.700000 | 35.433333 | 227 | 100.166667 | 39.150000 | 228 | 110.300000 | 38.833333 |
| 229 | 110.116667 | 37.116667 | 230 | 108.800000 | 37.600000 | 231 | 110.166667 | 37.766667 | 232 | 111.083333 | 39.033333 |
| 233 | 109.283333 | 37.950000 | 234 | 110.216667 | 37.500000 | 235 | 107.600000 | 37.583333 | 236 | 109.700000 | 38.233333 |
| 237 | 110.016667 | 37.600000 | 238 | 110.716667 | 37.516667 | 239 | 110.483333 | 38.016667 | 240 | 109.483333 | 36.600000 |
| 241 | 108.766667 | 36.866667 | 242 | 109.316667 | 36.883333 | 243 | 109.666667 | 37.133333 | 244 | 109.350000 | 36.266667 |
| 245 | 108.183333 | 36.833333 | 246 | 109.100000 | 35.400000 | 247 | 110.083333 | 36.866667 | 248 | 110.066667 | 36.583333 |
| 249 | 108.833333 | 34.250000 | 250 | 108.166667 | 36.933333 | 251 | 109.816667 | 35.600000 | 252 | 109.500000 | 35.816667 |
| 253 | 110.166667 | 36.033333 | 254 | 109.266667 | 35.583333 | 255 | 109.383333 | 36.000000 | 256 | 107.650000 | 34.450000 |
| 257 | 109.933333 | 34.800000 | 258 | 109.583333 | 34.950000 | 259 | 110.433333 | 35.466667 | 260 | 108.483333 | 34.500000 |
| 261 | 108.233333 | 34.516667 | 262 | 108.200000 | 34.283333 | 263 | 107.900000 | 34.350000 | 264 | 109.500000 | 34.500000 |
| 265 | 107.383333 | 34.516667 | 266 | 107.133333 | 34.350000 | 267 | 110.133333 | 34.083333 | 268 | 109.950000 | 33.916667 |
| 269 | 110.300000 | 33.700000 | 270 | 109.216667 | 34.366667 | 271 | 109.916667 | 35.183333 | 272 | 108.933333 | 34.150000 |
| 273 | 108.700000 | 34.350000 | 274 | 108.216667 | 34.150000 | 275 | 108.783333 | 34.600000 | 276 | 110.066667 | 34.566667 |
| 277 | 109.083333 | 34.533333 | 278 | 109.166667 | 34.750000 | 279 | 110.150000 | 35.233333 | 280 | 109.766667 | 34.516667 |
| 281 | 109.500000 | 35.250000 | 282 | 108.600000 | 34.100000 | 283 | 108.450000 | 34.333333 | 284 | 107.316667 | 34.100000 |
| 285 | 110.850000 | 33.500000 | 286 | 109.866667 | 33.516667 | 287 | 112.500000 | 40.000000 | 288 | 112.750000 | 40.083333 |
| 289 | 113.333333 | 40.100000 | 290 | 112.416667 | 39.316667 | 291 | 114.266667 | 39.766667 | 292 | 112.266667 | 39.516667 |
| 293 | 113.766667 | 40.366667 | 294 | 113.166667 | 39.566667 | 295 | 113.700000 | 39.700000 | 296 | 112.750000 | 39.516667 |
| 297 | 114.050000 | 40.433333 | 298 | 113.416667 | 39.516667 | 299 | 113.250000 | 38.716667 | 300 | 113.283333 | 39.183333 |
| 301 | 112.050000 | 37.216667 | 302 | 112.950000 | 38.466667 | 303 | 112.183333 | 39.083333 | 304 | 111.833333 | 38.900000 |
| 305 | 111.483333 | 39.433333 | 306 | 111.933333 | 38.350000 | 307 | 112.750000 | 38.716667 | 308 | 112.933333 | 39.050000 |
| 309 | 112.300000 | 39.000000 | 310 | 111.566667 | 38.700000 | 311 | 111.133333 | 39.383333 | 312 | 112.183333 | 37.200000 |
| 313 | 112.733333 | 37.683333 | 314 | 111.783333 | 37.250000 | 315 | 112.550000 | 37.783333 | 316 | 111.200000 | 37.350000 |
| 317 | 112.983333 | 37.066667 | 318 | 111.000000 | 37.966667 | 319 | 111.100000 | 37.500000 | 320 | 113.533333 | 37.333333 |
| 321 | 113.333333 | 38.083333 | 322 | 113.566667 | 37.933333 | 323 | 111.766667 | 37.133333 | 324 | 113.366667 | 37.066667 |
| 325 | 113.633333 | 37.800000 | 326 | 111.800000 | 36.850000 | 327 | 112.333333 | 37.600000 | 328 | 113.683333 | 37.600000 |
| 329 | 112.550000 | 37.450000 | 330 | 112.333333 | 37.350000 | 331 | 113.166667 | 37.900000 | 332 | 112.666667 | 38.050000 |
| 333 | 111.916667 | 37.033333 | 334 | 112.183333 | 37.550000 | 335 | 112.000000 | 37.416667 | 336 | 111.116667 | 38.466667 |
| 337 | 111.233333 | 37.883333 | 338 | 111.666667 | 38.266667 | 339 | 111.166667 | 36.983333 | 340 | 111.133333 | 37.533333 |
| 341 | 110.883333 | 37.433333 | 342 | 112.916667 | 35.783333 | 343 | 112.833333 | 35.516667 | 344 | 112.183333 | 35.700000 |
| 345 | 113.266667 | 35.766667 | 346 | 113.416667 | 36.200000 | 347 | 113.250000 | 36.083333 | 348 | 113.383333 | 36.500000 |
| 349 | 112.850000 | 36.833333 | 350 | 112.166667 | 36.500000 | 351 | 112.416667 | 35.483333 | 352 | 113.066667 | 36.200000 |
| 353 | 113.216667 | 36.333333 | 354 | 112.883333 | 36.316667 | 355 | 113.033333 | 36.516667 | 356 | 112.683333 | 36.766667 |
| 357 | 112.866667 | 36.116667 | 358 | 112.666667 | 35.500000 | 359 | 110.933333 | 36.700000 | 360 | 115.816667 | 40.900000 |
| 361 | 115.050000 | 40.616667 | 362 | 115.216667 | 40.366667 | 363 | 114.566667 | 39.833333 | 364 | 114.883333 | 40.833333 |
| 365 | 114.716667 | 40.750000 | 366 | 115.500000 | 40.400000 | 367 | 114.416667 | 40.666667 | 368 | 115.283333 | 40.950000 |
| 369 | 114.166667 | 40.116667 | 370 | 115.966667 | 40.466667 | 371 | 114.683333 | 41.250000 | 372 | 113.566667 | 41.583333 |
| 373 | 114.600000 | 41.850000 | 374 | 117.766667 | 41.950000 | 375 | 117.716667 | 41.300000 | 376 | 117.950000 | 41.000000 |
| 377 | 118.700000 | 41.000000 | 378 | 116.633333 | 41.216667 | 379 | 119.933333 | 40.416667 | 380 | 117.316667 | 40.916667 |
| 381 | 117.483333 | 40.400000 | 382 | 118.683333 | 39.733333 | 383 | 117.716667 | 39.750000 | 384 | 118.300000 | 40.150000 |
| 385 | 118.866667 | 39.883333 | 386 | 119.233333 | 39.883333 | 387 | 118.050000 | 39.800000 | 388 | 119.150000 | 39.700000 |
| 389 | 118.683333 | 39.500000 | 390 | 118.116667 | 39.550000 | 391 | 114.433333 | 38.066667 | 392 | 117.566667 | 41.000000 |
| 393 | 118.716667 | 40.000000 | 394 | 117.066667 | 39.983333 | 395 | 116.466667 | 38.866667 | 396 | 116.983333 | 39.766667 |
| 397 | 116.583333 | 38.733333 | 398 | 117.100000 | 39.400000 | 399 | 116.916667 | 38.933333 | 400 | 117.300000 | 39.716667 |
| 401 | 116.700000 | 39.500000 | 402 | 116.500000 | 39.316667 | 403 | 117.250000 | 40.000000 | 404 | 116.600000 | 40.133333 |
| 405 | 116.000000 | 39.700000 | 406 | 116.633333 | 40.300000 | 407 | 116.216667 | 40.216667 | 408 | 117.116667 | 40.133333 |
| 409 | 116.650000 | 39.900000 | 410 | 116.333333 | 39.733333 | 411 | 116.283333 | 39.866667 | 412 | 115.316667 | 38.950000 |
| 413 | 115.133333 | 38.833333 | 414 | 114.700000 | 38.616667 | 415 | 115.000000 | 38.516667 | 416 | 115.700000 | 39.383333 |
| 417 | 114.983333 | 38.750000 | 418 | 114.183333 | 38.833333 | 419 | 115.516667 | 38.850000 | 420 | 115.483333 | 39.333333 |
| 421 | 114.683333 | 39.350000 | 422 | 115.766667 | 39.266667 | 423 | 115.450000 | 38.450000 | 424 | 116.083333 | 38.966667 |
| 425 | 115.966667 | 39.483333 | 426 | 115.866667 | 39.316667 | 427 | 115.650000 | 39.016667 | 428 | 115.316667 | 38.416667 |
| 429 | 115.500000 | 38.550000 | 430 | 115.133333 | 38.700000 | 431 | 115.866667 | 39.033333 | 432 | 115.766667 | 38.683333 |
| 433 | 114.516667 | 37.750000 | 434 | 114.616667 | 37.600000 | 435 | 115.216667 | 37.916667 | 436 | 114.833333 | 38.033333 |
| 437 | 114.750000 | 37.833333 | 438 | 114.416667 | 38.033333 | 439 | 114.166667 | 38.000000 | 440 | 114.300000 | 38.083333 |
| 441 | 114.583333 | 38.150000 | 442 | 115.033333 | 38.033333 | 443 | 114.550000 | 38.450000 | 444 | 114.366667 | 38.300000 |
| 445 | 114.816667 | 38.100000 | 446 | 114.683333 | 38.350000 | 447 | 114.633333 | 37.883333 | 448 | 115.633333 | 37.716667 |
| 449 | 115.583333 | 37.566667 | 450 | 115.550000 | 38.000000 | 451 | 115.900000 | 37.783333 | 452 | 115.516667 | 38.233333 |
| 453 | 115.883333 | 37.333333 | 454 | 116.250000 | 37.683333 | 455 | 116.066667 | 37.800000 | 456 | 115.983333 | 38.033333 |
| 457 | 115.733333 | 38.233333 | 458 | 115.750000 | 37.500000 | 459 | 114.683333 | 37.000000 | 460 | 114.483333 | 36.766667 |
| 461 | 114.933333 | 36.766667 | 462 | 114.366667 | 36.350000 | 463 | 114.500000 | 36.583333 | 464 | 114.916667 | 36.350000 |
| 465 | 113.666667 | 36.566667 | 466 | 114.183333 | 36.700000 | 467 | 115.150000 | 36.800000 | 468 | 114.600000 | 36.350000 |
| 469 | 114.950000 | 36.483333 | 470 | 114.800000 | 36.550000 | 471 | 116.083333 | 38.700000 | 472 | 115.833333 | 38.433333 |
| 473 | 116.250000 | 38.033333 | 474 | 117.233333 | 38.033333 | 475 | 116.916667 | 38.333333 | 476 | 116.050000 | 38.416667 |
| 477 | 117.433333 | 38.450000 | 478 | 116.116667 | 38.183333 | 479 | 116.533333 | 37.883333 | 480 | 116.433333 | 37.633333 |
| 481 | 115.116667 | 35.900000 | 482 | 114.500000 | 35.583333 | 483 | 114.500000 | 35.183333 | 484 | 114.916667 | 35.933333 |
| 485 | 113.983333 | 36.066667 | 486 | 114.400000 | 35.883333 | 487 | 114.333333 | 36.116667 | 488 | 114.533333 | 35.666667 |
| 489 | 115.016667 | 35.700000 | 490 | 114.200000 | 35.600000 | 491 | 115.200000 | 36.083333 | 492 | 113.633333 | 34.783333 |
| 493 | 112.800000 | 34.916667 | 494 | 112.633333 | 35.083333 | 495 | 113.816667 | 35.450000 | 496 | 113.883333 | 35.316667 |
| 497 | 114.400000 | 35.033333 | 498 | 114.183333 | 35.133333 | 499 | 113.950000 | 35.050000 | 500 | 114.066667 | 35.416667 |
| 501 | 113.383333 | 35.100000 | 502 | 113.383333 | 35.333333 | 503 | 113.083333 | 34.933333 | 504 | 114.333333 | 35.083333 |
| 505 | 112.916667 | 35.116667 | 506 | 113.066667 | 35.166667 | 507 | 114.266667 | 33.266667 | 508 | 113.266667 | 35.233333 |
| 509 | 113.650000 | 35.266667 | 510 | 115.133333 | 34.650000 | 511 | 112.833333 | 32.683333 | 512 | 113.716667 | 34.483333 |
| 513 | 113.033333 | 34.450000 | 514 | 115.483333 | 33.800000 | 515 | 111.500000 | 33.300000 | 516 | 114.350000 | 33.000000 |
| 517 | 114.733333 | 32.350000 | 518 | 115.300000 | 34.066667 | 519 | 115.800000 | 34.450000 | 520 | 114.883333 | 33.450000 |
| 521 | 115.316667 | 34.433333 | 522 | 115.883333 | 34.383333 | 523 | 115.066667 | 34.416667 | 524 | 114.366667 | 34.783333 |
| 525 | 113.366667 | 34.783333 | 526 | 112.533333 | 32.983333 | 527 | 116.400000 | 33.933333 | 528 | 114.200000 | 34.416667 |
| 529 | 112.983333 | 34.750000 | 530 | 113.316667 | 32.716667 | 531 | 114.066667 | 33.016667 | 532 | 114.800000 | 34.816667 |
| 533 | 112.400000 | 32.516667 | 534 | 115.083333 | 35.283333 | 535 | 114.066667 | 32.116667 | 536 | 113.383333 | 34.533333 |
| 537 | 114.783333 | 34.550000 | 538 | 116.050000 | 34.233333 | 539 | 114.016667 | 33.150000 | 540 | 114.850000 | 33.716667 |
| 541 | 114.666667 | 32.966667 | 542 | 114.850000 | 34.066667 | 543 | 112.583333 | 33.033333 | 544 | 115.066667 | 33.383333 |
| 545 | 114.300000 | 32.616667 | 546 | 114.050000 | 32.800000 | 547 | 115.200000 | 33.516667 | 548 | 113.000000 | 33.266667 |
| 549 | 112.083333 | 32.700000 | 550 | 117.750000 | 35.900000 | 551 | 111.500000 | 33.133333 | 552 | 111.850000 | 33.050000 |
| 553 | 113.416667 | 32.366667 | 554 | 114.016667 | 34.666667 | 555 | 114.016667 | 33.400000 | 556 | 112.233333 | 33.050000 |
| 557 | 112.433333 | 33.483333 | 558 | 115.383333 | 31.783333 | 559 | 112.416667 | 34.416667 | 560 | 110.033333 | 34.050000 |
| 561 | 111.750000 | 34.766667 | 562 | 111.600000 | 33.783333 | 563 | 112.783333 | 34.733333 | 564 | 112.433333 | 34.650000 |
| 565 | 112.083333 | 34.150000 | 566 | 112.433333 | 34.816667 | 567 | 112.466667 | 34.150000 | 568 | 112.833333 | 33.183333 |
| 569 | 111.183333 | 34.766667 | 570 | 112.183333 | 34.500000 | 571 | 111.633333 | 34.383333 | 572 | 112.816667 | 34.716667 |
| 573 | 113.050000 | 33.866667 | 574 | 113.450000 | 34.150000 | 575 | 113.566667 | 33.400000 | 576 | 114.383333 | 34.050000 |
| 577 | 114.633333 | 33.633333 | 578 | 114.200000 | 34.083333 | 579 | 113.850000 | 34.016667 | 580 | 114.000000 | 33.583333 |
| 581 | 112.900000 | 33.733333 | 582 | 113.950000 | 33.816667 | 583 | 114.516667 | 33.783333 | 584 | 113.466667 | 33.850000 |
| 585 | 113.500000 | 33.850000 | 586 | 113.800000 | 34.200000 | 587 | 113.350000 | 33.633333 | 588 | 113.283333 | 33.733333 |
| 589 | 114.716667 | 33.500000 | 590 | 113.200000 | 33.966667 | 591 | 117.983333 | 37.466667 | 592 | 115.700000 | 36.833333 |
| 593 | 117.200000 | 36.966667 | 594 | 117.733333 | 36.900000 | 595 | 117.183333 | 37.716667 | 596 | 121.033333 | 36.783333 |
| 597 | 119.450000 | 35.433333 | 598 | 118.400000 | 37.050000 | 599 | 118.083333 | 36.950000 | 600 | 117.000000 | 35.400000 |
| 601 | 117.066667 | 36.683333 | 602 | 121.600000 | 37.400000 | 603 | 120.850000 | 37.300000 | 604 | 122.383333 | 37.166667 |
| 605 | 117.133333 | 35.116667 | 606 | 118.550000 | 36.500000 | 607 | 119.183333 | 36.433333 | 608 | 118.116667 | 37.150000 |
| 609 | 118.016667 | 37.383333 | 610 | 118.350000 | 35.050000 | 611 | 117.150000 | 36.166667 | 612 | 116.250000 | 36.333333 |
| 613 | 116.450000 | 35.933333 | 614 | 116.266667 | 36.866667 | 615 | 119.183333 | 35.733333 | 616 | 118.333333 | 34.600000 |
| 617 | 118.833333 | 35.583333 | 618 | 119.916667 | 36.750000 | 619 | 120.466667 | 36.383333 | 620 | 115.433333 | 36.483333 |
| 621 | 121.266667 | 37.500000 | 622 | 116.766667 | 36.183333 | 623 | 119.933333 | 37.183333 | 624 | 118.116667 | 37.700000 |
| 625 | 119.750000 | 36.366667 | 626 | 115.833333 | 36.000000 | 627 | 116.950000 | 36.683333 | 628 | 120.000000 | 35.883333 |
| 629 | 117.600000 | 37.733333 | 630 | 116.316667 | 37.433333 | 631 | 120.450000 | 36.383333 | 632 | 116.333333 | 35.400000 |
| 633 | 116.850000 | 37.183333 | 634 | 117.616667 | 35.500000 | 635 | 120.683333 | 36.900000 | 636 | 122.033333 | 37.183333 |
| 637 | 117.650000 | 36.250000 | 638 | 117.750000 | 36.750000 | 639 | 119.183333 | 36.750000 | 640 | 116.466667 | 35.716667 |
| 641 | 120.516667 | 37.650000 | 642 | 115.500000 | 35.083333 | 643 | 121.500000 | 36.916667 | 644 | 118.616667 | 35.783333 |
| 645 | 118.333333 | 36.833333 | 646 | 118.483333 | 35.550000 | 647 | 116.750000 | 36.783333 | 648 | 116.083333 | 35.400000 |
| 649 | 116.966667 | 35.600000 | 650 | 116.433333 | 35.133333 | 651 | 117.950000 | 35.250000 | 652 | 117.516667 | 36.716667 |
| 653 | 116.100000 | 35.800000 | 654 | 119.100000 | 36.716667 | 655 | 117.150000 | 37.166667 | 656 | 118.233333 | 37.483333 |
| 657 | 117.266667 | 35.650000 | 658 | 116.300000 | 35.066667 | 659 | 119.416667 | 36.833333 | 660 | 116.083333 | 34.783333 |
| 661 | 120.750000 | 37.816667 | 662 | 120.383333 | 37.350000 | 663 | 115.533333 | 34.816667 | 664 | 116.800000 | 35.766667 |
| 665 | 115.800000 | 36.133333 | 666 | 117.116667 | 34.766667 | 667 | 116.500000 | 36.233333 | 668 | 117.916667 | 35.716667 |
| 669 | 118.833333 | 36.700000 | 670 | 118.816667 | 35.183333 | 671 | 115.950000 | 35.600000 | 672 | 118.733333 | 36.883333 |
| 673 | 115.950000 | 36.916667 | 674 | 115.400000 | 35.200000 | 675 | 116.366667 | 37.166667 | 676 | 117.583333 | 37.650000 |
| 677 | 118.166667 | 36.183333 | 678 | 117.800000 | 34.766667 | 679 | 116.250000 | 36.583333 | 680 | 116.633333 | 36.933333 |
| 681 | 118.466667 | 36.700000 | 682 | 119.416667 | 35.983333 | 683 | 108.100000 | 34.900000 | 684 | 115.500000 | 35.800000 |
| 685 | 120.483333 | 36.866667 | 686 | 115.966667 | 36.433333 | 687 | 116.216667 | 36.583333 | 688 | 120.000000 | 36.266667 |
| 689 | 118.100000 | 34.900000 | 690 | 115.883333 | 34.950000 | 691 | 115.666667 | 36.250000 | 692 | 121.383333 | 37.533333 |
| 693 | 115.516667 | 35.550000 | 694 | 116.833333 | 40.366667 | 695 | 116.100000 | 39.933333 | 696 | 115.483333 | 36.850000 |
| 697 | 114.500000 | 37.433333 | 698 | 118.900000 | 39.416667 | 699 | 118.150000 | 39.700000 | 700 | 117.816667 | 39.333333 |
| 701 | 116.600000 | 39.533333 | 702 | 116.300000 | 39.433333 | 703 | 116.383333 | 39.116667 | 704 | 115.566667 | 38.483333 |
| 705 | 114.133333 | 38.033333 | 706 | 114.933333 | 37.600000 | 707 | 115.233333 | 37.516667 | 708 | 115.366667 | 37.350000 |
| 709 | 114.500000 | 37.283333 | 710 | 114.766667 | 37.350000 | 711 | 115.033333 | 37.216667 | 712 | 114.666667 | 37.133333 |
| 713 | 114.383333 | 37.050000 | 714 | 115.133333 | 37.050000 | 715 | 115.250000 | 36.966667 | 716 | 114.716667 | 37.000000 |
| 717 | 115.016667 | 37.050000 | 718 | 115.650000 | 37.083333 | 719 | 114.500000 | 36.866667 | 720 | 116.066667 | 37.216667 |
| 721 | 119.150000 | 36.600000 | 722 | 117.850000 | 36.483333 | 723 | 115.666667 | 36.200000 | 724 | 112.700000 | 38.400000 |
| 725 | 111.500000 | 36.066667 | 726 | 111.466667 | 35.650000 | 727 | 110.450000 | 34.883333 | 728 | 115.100000 | 34.433333 |
| 729 | 109.100000 | 35.116667 | 730 | 117.233333 | 34.266667 | 731 | 117.283333 | 31.883333 | 732 | 120.133333 | 30.183333 |
| 733 | 121.416667 | 29.283333 | 734 | 117.483333 | 27.333333 | 735 | 109.650000 | 28.716667 | 736 | 109.733333 | 28.316667 |
| 737 | 109.600000 | 27.983333 | 738 | 110.383333 | 28.450000 | 739 | 114.400000 | 23.083333 | 740 | 110.450000 | 19.233333 |
| 741 | 110.350000 | 20.000000 | 742 | 110.733333 | 19.616667 | 743 | 109.566667 | 19.516667 | 744 | 106.716667 | 26.583333 |
| 745 | 107.500000 | 25.000000 | 746 | 106.616667 | 23.900000 | 747 | 102.750000 | 25.016667 | 748 | 100.800000 | 22.000000 |
| 749 | 117.216667 | 34.233333 | 750 | 117.950000 | 34.300000 | 751 | 118.333333 | 34.366667 | 752 | 117.950000 | 28.433333 |
| 753 | 116.683333 | 29.000000 | 754 | 117.133333 | 28.966667 | 755 | 117.216667 | 28.300000 | 756 | 117.866667 | 29.266667 |
| 757 | 117.066667 | 28.700000 | 758 | 117.700000 | 28.316667 | 759 | 118.200000 | 28.433333 | 760 | 116.266667 | 28.366667 |
| 761 | 115.783333 | 29.466667 | 762 | 116.550000 | 29.900000 | 763 | 116.216667 | 29.750000 | 764 | 116.183333 | 29.100000 |
| 765 | 114.683333 | 27.816667 | 766 | 115.100000 | 29.266667 | 767 | 116.033333 | 29.283333 | 768 | 115.500000 | 29.666667 |
| 769 | 110.816667 | 32.850000 | 770 | 112.750000 | 32.150000 | 771 | 111.666667 | 32.266667 | 772 | 111.833333 | 31.783333 |
| 773 | 111.250000 | 31.866667 | 774 | 111.666667 | 32.416667 | 775 | 113.383333 | 31.716667 | 776 | 111.333333 | 30.766667 |
| 777 | 110.683333 | 31.000000 | 778 | 111.666667 | 31.066667 | 779 | 110.683333 | 30.150000 | 780 | 111.750000 | 30.433333 |
| 781 | 111.183333 | 30.466667 | 782 | 111.450000 | 30.400000 | 783 | 111.783333 | 30.833333 | 784 | 110.766667 | 31.233333 |
| 785 | 110.416667 | 31.033333 | 786 | 109.633333 | 30.600000 | 787 | 109.466667 | 30.283333 | 788 | 109.400000 | 29.516667 |
| 789 | 112.883333 | 30.416667 | 790 | 112.150000 | 30.066667 | 791 | 113.433333 | 29.816667 | 792 | 114.066667 | 31.566667 |
| 793 | 114.366667 | 30.866667 | 794 | 113.816667 | 30.666667 | 795 | 115.416667 | 30.233333 | 796 | 115.933333 | 30.066667 |
| 797 | 115.666667 | 30.733333 | 798 | 115.033333 | 31.183333 | 799 | 114.950000 | 30.083333 | 800 | 115.200000 | 29.833333 |
| 801 | 114.483333 | 29.600000 | 802 | 114.000000 | 29.500000 | 803 | 109.183333 | 27.716667 | 804 | 108.750000 | 27.216667 |
| 805 | 108.416667 | 27.050000 | 806 | 108.133333 | 27.050000 | 807 | 107.766667 | 26.850000 | 808 | 109.200000 | 26.916667 |
| 809 | 108.533333 | 26.950000 | 810 | 108.583333 | 26.650000 | 811 | 108.316667 | 26.666667 | 812 | 107.983333 | 26.600000 |
| 813 | 107.566667 | 26.483333 | 814 | 108.133333 | 26.383333 | 815 | 107.800000 | 26.200000 | 816 | 109.183333 | 26.666667 |
| 817 | 109.133333 | 26.233333 | 818 | 108.900000 | 25.750000 | 819 | 103.033333 | 25.333333 | 820 | 102.500000 | 25.216667 |
| 821 | 103.400000 | 23.366667 | 822 | 101.716667 | 23.433333 | 823 | 126.650000 | 51.716667 | 824 | 125.066667 | 45.516667 |
| 825 | 125.966667 | 46.066667 | 826 | 119.750000 | 49.216667 | 827 | 119.683333 | 49.216667 | 828 | 130.350000 | 42.866667 |
| 829 | 129.833333 | 42.966667 | 830 | 128.916667 | 43.116667 | 831 | 129.000000 | 42.550000 | 832 | 120.066667 | 43.900000 |
| 833 | 119.366667 | 43.966667 | 834 | 122.266667 | 43.750000 | 835 | 113.183333 | 41.450000 | 836 | 113.000000 | 39.833333 |
| 837 | 111.766667 | 38.066667 | 838 | 111.566667 | 36.683333 | 839 | 111.716667 | 36.583333 | 840 | 111.100000 | 36.400000 |
| 841 | 111.666667 | 36.250000 | 842 | 111.916667 | 36.266667 | 843 | 112.250000 | 36.166667 | 844 | 111.833333 | 35.966667 |
| 845 | 111.450000 | 35.883333 | 846 | 111.716667 | 35.733333 | 847 | 111.366667 | 35.733333 | 848 | 110.633333 | 36.766667 |
| 849 | 110.750000 | 36.466667 | 850 | 110.683333 | 36.100000 | 851 | 110.850000 | 35.966667 | 852 | 111.566667 | 35.483333 |
| 853 | 111.833333 | 35.333333 | 854 | 111.200000 | 35.333333 | 855 | 111.216667 | 35.133333 | 856 | 111.216667 | 34.866667 |
| 857 | 111.216667 | 35.616667 | 858 | 110.900000 | 35.583333 | 859 | 110.983333 | 35.016667 | 860 | 110.833333 | 35.416667 |
| 861 | 110.716667 | 35.150000 | 862 | 110.716667 | 34.700000 | 863 | 108.983333 | 34.933333 | 864 | 108.333333 | 35.116667 |
| 865 | 108.033333 | 35.033333 | 866 | 108.216667 | 34.733333 | 867 | 107.783333 | 34.683333 | 868 | 107.800000 | 35.216667 |
| 869 | 108.816667 | 34.550000 | 870 | 106.850000 | 34.883333 | 871 | 109.000000 | 33.666667 | 872 | 108.900000 | 32.300000 |
| 873 | 109.516667 | 31.883333 | 874 | 106.883333 | 33.533333 | 875 | 106.133333 | 33.333333 | 876 | 106.250000 | 32.833333 |
| 877 | 103.866667 | 35.383333 | 878 | 103.183333 | 35.616667 | 879 | 103.083333 | 38.616667 | 880 | 102.900000 | 37.483333 |
| 881 | 100.816667 | 38.433333 | 882 | 106.333333 | 38.533333 | 883 | 105.900000 | 36.966667 | 884 | 105.633333 | 36.566667 |
| 885 | 106.283333 | 36.000000 | 886 | 101.833333 | 36.683333 | 887 | 102.266667 | 35.100000 | 888 | 102.550000 | 35.833333 |
| 889 | 102.800000 | 36.316667 | 890 | 102.500000 | 36.500000 | 891 | 101.766667 | 36.616667 | 892 | 101.583333 | 36.500000 |
| 893 | 118.466667 | 40.550000 | 894 | 114.200000 | 38.316667 | 895 | 114.366667 | 37.650000 | 896 | 118.533333 | 37.600000 |
| 897 | 117.383333 | 37.766667 | 898 | 116.566667 | 37.350000 | 899 | 118.000000 | 36.833333 | 900 | 117.533333 | 34.850000 |
| 901 | 117.733333 | 34.466667 | 902 | 112.150000 | 34.716667 | 903 | 123.966667 | 44.283333 | 904 | 106.533333 | 38.900000 |
| 905 | 106.250000 | 38.266667 | 906 | 106.216667 | 38.000000 | 907 | 106.066667 | 38.016667 | 908 | 105.716667 | 35.966667 |
| 909 | 103.733333 | 29.550000 | 910 | 116.583333 | 38.066667 | 911 | 116.833333 | 38.333333 | 912 | 115.866667 | 35.983333 |
| 913 | 120.316667 | 37.616667 | 914 | 116.816667 | 35.550000 | 915 | 117.166667 | 35.083333 | 916 | 110.833333 | 36.983333 |
| 917 | 108.300000 | 33.316667 | 918 | 109.366667 | 32.850000 | 919 | 110.116667 | 32.816667 | 920 | 109.033333 | 32.716667 |
| 921 | 109.350000 | 32.383333 | 922 | 106.050000 | 35.216667 | 923 | 101.083333 | 38.800000 | 924 | 110.450000 | 31.100000 |
| 925 | 110.733333 | 32.066667 | 926 | 109.683333 | 32.316667 | 927 | 109.150000 | 29.683333 | 928 | 109.383333 | 29.983333 |
| 929 | 110.033333 | 29.883333 | 930 | 116.683333 | 28.683333 | 931 | 109.883333 | 31.066667 | 932 | 109.633333 | 31.400000 |
| 933 | 109.516667 | 31.050000 | 934 | 103.266667 | 25.566667 | 935 | 102.466667 | 25.550000 | 936 | 102.800000 | 24.900000 |
| 937 | 102.583333 | 24.600000 | 938 | 102.483333 | 24.933333 | 939 | 102.400000 | 25.533333 | 940 | 100.750000 | 26.683333 |
| 941 | 100.583333 | 25.483333 | 942 | 103.250000 | 23.700000 | 943 | 107.233333 | 26.583333 | 944 | 106.466667 | 26.566667 |
| 945 | 106.233333 | 27.466667 | 946 | 105.283333 | 27.300000 | 947 | 104.866667 | 26.583333 | 948 | 105.116667 | 25.983333 |
| 949 | 104.233333 | 33.266667 | 950 | 107.233333 | 31.933333 | 951 | 106.283333 | 32.216667 | 952 | 103.133333 | 28.316667 |
| 953 | 102.850000 | 28.016667 | 954 | 110.333333 | 20.033333 | 955 | 110.333333 | 19.700000 | 956 | 110.000000 | 19.733333 |
| 957 | 106.733333 | 25.416667 | 958 | 106.083333 | 25.166667 | 959 | 109.683333 | 19.900000 | 960 | 106.200000 | 25.700000 |
| 961 | 110.400000 | 18.800000 | 962 | 109.450000 | 19.100000 | 963 | 109.050000 | 19.266667 | 964 | 108.633333 | 19.033333 |
| 965 | 105.816667 | 24.983333 | 966 | 107.516667 | 26.266667 | 967 | 109.650000 | 18.750000 | 968 | 110.033333 | 18.500000 |
| 969 | 109.516667 | 18.250000 | 970 | 108.483333 | 28.550000 | 971 | 108.116667 | 28.250000 | 972 | 108.383333 | 28.000000 |
| 973 | 109.183333 | 28.150000 | 974 | 108.233333 | 27.566667 | 975 | 107.883333 | 28.516667 | 976 | 106.200000 | 28.316667 |
| 977 | 106.400000 | 27.816667 | 978 | 105.533333 | 27.116667 | 979 | 106.650000 | 26.133333 | 980 | 111.150000 | 25.483333 |
| 981 | 106.566667 | 24.800000 | 982 | 105.333333 | 24.783333 | 983 | 105.833333 | 23.416667 | 984 | 106.416667 | 23.133333 |
| 985 | 110.333333 | 22.700000 | 986 | 108.333333 | 22.150000 | 987 | 107.683333 | 23.166667 | 988 | 107.233333 | 25.016667 |
| 989 | 107.050000 | 24.516667 | 990 | 110.550000 | 22.850000 | 991 | 102.066667 | 25.150000 | 992 | 100.416667 | 21.916667 |
| 993 | 108.233333 | 27.933333 | 994 | 106.833333 | 27.533333 | 995 | 104.650000 | 25.783333 | 996 | 108.500000 | 25.850000 |
| 997 | 107.500000 | 26.700000 | 998 | 107.866667 | 25.983333 | 999 | 107.550000 | 25.833333 | 1000 | 107.316667 | 25.833333 |
| 1001 | 107.883333 | 25.416667 | 1002 | 106.966667 | 27.066667 | 1003 | 106.733333 | 27.100000 | 1004 | 106.600000 | 26.850000 |
| 1005 | 106.266667 | 26.416667 | 1006 | 105.916667 | 26.233333 | 1007 | 105.750000 | 26.300000 | 1008 | 105.766667 | 26.050000 |
| 1009 | 105.616667 | 25.950000 | 1010 | 104.950000 | 25.783333 | 1011 | 105.183333 | 25.433333 | 1012 | 105.633333 | 25.400000 |
| 1013 | 105.466667 | 25.116667 | 1014 | 104.900000 | 25.083333 | 1015 | 99.287170 | 27.177166 | 1016 | 104.702829 | 23.125462 |
| 1017 | 121.642083 | 25.000000 | 1018 | 116.346461 | 40.016934 | 1019 | 116.077021 | 40.806404 | 1020 | 108.866670 | 34.250000 |
| 1021 | 116.000000 | 40.116670 | 1022 | 116.000000 | 39.916670 | 1023 | 110.670000 | 31.750000 | 1024 | 86.870000 | 42.270000 |
| 1025 | 107.100000 | 29.160000 | 1026 | 102.480000 | 34.580000 | 1027 | 100.800000 | 22.020000 | 1028 | 110.980000 | 37.950000 |
| 1029 | 114.130000 | 24.350000 | 1030 | 119.520000 | 25.970000 | 1031 | 99.980000 | 31.620000 | 1032 | 91.140000 | 29.650000 |
| 1033 | 118.130000 | 30.300000 | 1034 | 119.670000 | 29.800000 | 1035 | 104.370000 | 33.780000 | 1036 | 114.380000 | 37.670000 |
| 1037 | 110.200000 | 25.230000 | 1038 | 109.470000 | 30.300000 | 1039 | 104.680000 | 32.950000 | 1040 | 108.070000 | 26.380000 |
| 1041 | 122.100000 | 30.020000 | 1042 | 115.500000 | 39.350000 | 1043 | 86.220000 | 44.300000 | 1044 | 112.950000 | 25.400000 |
| 1045 | 118.150000 | 24.730000 | 1046 | 107.750000 | 34.280000 | 1047 | 109.700000 | 18.630000 | 1048 | 81.320000 | 43.920000 |
| 1049 | 114.520000 | 37.300000 | 1050 | 110.920000 | 23.380000 | 1051 | 102.230000 | 29.920000 | 1052 | 106.400000 | 29.800000 |
| 1053 | 113.100000 | 41.030000 | 1054 | 114.300000 | 30.570000 | 1055 | 110.330000 | 31.050000 | 1056 | 109.320000 | 34.150000 |
| 1057 | 109.730000 | 30.600000 | 1058 | 86.400000 | 42.320000 | 1059 | 110.450000 | 34.870000 | 1060 | 103.250000 | 27.700000 |
| 1061 | 131.870000 | 45.550000 | 1062 | 109.470000 | 31.020000 | 1063 | 117.330000 | 27.550000 | 1064 | 119.720000 | 30.230000 |
| 1065 | 121.720000 | 29.950000 | 1066 | 119.130000 | 28.080000 | 1067 | 119.050000 | 27.620000 | 1068 | 114.700000 | 41.150000 |
| 1069 | 118.950000 | 40.400000 | 1070 | 115.980000 | 29.680000 | 1071 | 104.520000 | 28.430000 | 1072 | 104.920000 | 33.400000 |
| 1073 | 117.150000 | 39.130000 | 1074 | 104.180000 | 36.550000 | 1075 | 108.720000 | 39.830000 | 1076 | 116.180000 | 40.210000 |
| 1077 | 106.670000 | 33.150000 | 1078 | 122.750000 | 47.980000 | 1079 | 127.280000 | 42.330000 | 1080 | 101.880000 | 25.700000 |
| 1081 | 110.670000 | 25.620000 | 1082 | 109.850000 | 29.000000 | 1083 | 115.400000 | 30.780000 | 1084 | 86.085507 | 44.312423 |
| 1085 | 114.480000 | 38.070000 | 1086 | 104.140000 | 36.540000 | 1087 | 120.170000 | 30.270000 | 1088 | 112.850000 | 35.490000 |
| 1089 | 119.050000 | 39.620000 | 1090 | 113.870000 | 35.340000 | 1091 | 114.550000 | 34.710000 | 1092 | 117.090000 | 40.500000 |
| 1093 | 128.510000 | 42.780000 | 1094 | 95.330000 | 29.320000 | 1095 | 113.920000 | 35.030000 | 1096 | 107.970000 | 26.580000 |
| 1097 | 103.750000 | 36.070000 | 1098 | 113.430000 | 38.790000 | 1099 | 119.000000 | 39.360000 | 1100 | 119.360000 | 30.190000 |
| 1101 | 98.348100 | 27.736900 | 1102 | 128.000000 | 48.000000 | 1103 | 129.430000 | 44.380000 | 1104 | 126.500000 | 43.633000 |
| 1105 | 121.778332 | 41.363609 | 1106 | 117.280555 | 31.863888 | 1107 | 119.342114 | 32.970736 | 1108 | 99.706013 | 27.823080 |
| 1109 | 113.045465 | 23.688233 | 1110 | 113.184435 | 24.813781 | 1111 | 111.000731 | 22.008084 | 1112 | 103.398589 | 29.496119 |
| 1113 | 109.511923 | 18.252625 | 1114 | 119.578275 | 29.843296 | 1115 | 113.884138 | 35.246355 | 1116 | 110.340757 | 31.042327 |
| 1117 | 109.411179 | 31.054680 | 1118 | 107.545509 | 33.222857 | 1119 | 113.341527 | 23.127041 | 1120 | 110.050532 | 25.255089 |
| 1121 | 110.351669 | 25.175621 | 1122 | 110.585686 | 25.601680 | 1123 | 108.487812 | 22.758186 | 1124 | 120.976081 | 29.144543 |
| 1125 | 113.987821 | 24.399048 | 1126 | 110.040007 | 25.742153 | 1127 | 108.283333 | 41.033333 | 1128 | 118.879583 | 42.297433 |
| 1129 | 112.966667 | 37.700000 | 1130 | 112.466667 | 37.200000 | 1131 | 113.133333 | 36.200000 | 1132 | 111.790000 | 37.260000 |
| 1133 | 113.690000 | 39.300000 | 1134 | 113.083333 | 37.850000 | 1135 | 112.860000 | 37.810000 | 1136 | 112.720000 | 37.610000 |
| 1137 | 112.890000 | 35.830000 | 1138 | 112.680000 | 36.740000 | 1139 | 112.416667 | 37.416667 | 1140 | 112.590000 | 37.420000 |
| 1141 | 112.916667 | 37.700000 | 1142 | 113.140833 | 36.215833 | 1143 | 112.483333 | 37.383333 | 1144 | 112.483333 | 36.416667 |
| 1145 | 111.244750 | 25.659194 | 1146 | 110.170556 | 28.358694 | 1147 | 115.972958 | 43.631242 | 1148 | 114.630003 | 31.245989 |
| 1149 | 116.927394 | 35.629456 | 1150 | 117.520308 | 35.711553 | 1151 | 116.354067 | 29.318014 | 1152 | 121.778136 | 37.321467 |
| 1153 | 109.065136 | 34.450903 | 1154 | 117.590500 | 44.604269 | 1155 | 114.358889 | 27.256111 | 1156 | 109.849056 | 28.693056 |
| 1157 | 118.700000 | 39.983333 | 1158 | 114.583333 | 39.850000 | 1159 | 114.383333 | 36.366667 | 1160 | 111.800000 | 38.066667 |
| 1161 | 113.966667 | 35.050000 | 1162 | 114.166667 | 35.200000 | 1163 | 113.816667 | 36.066667 | 1164 | 124.016667 | 45.016667 |
| 1165 | 123.833333 | 44.816667 | 1166 | 123.183333 | 47.433333 | 1167 | 126.283333 | 46.266667 | 1168 | 111.683333 | 39.916667 |
| 1169 | 107.650000 | 35.733333 | 1170 | 106.333333 | 38.100000 | 1171 | 110.500000 | 38.850000 | 1172 | 110.183333 | 37.750000 |
| 1173 | 117.533333 | 36.683333 | 1174 | 117.866667 | 36.800000 | 1175 | 118.633333 | 35.783333 | 1176 | 87.550000 | 44.166667 |
| 1177 | 113.100000 | 36.183333 | 1178 | 112.066667 | 40.033333 | 1179 | 109.383333 | 37.966667 | 1180 | 113.800000 | 35.300000 |
| 1181 | 112.400000 | 34.600000 | 1182 | 112.338300 | 36.183900 | 1183 | 125.214100 | 49.166100 | 1184 | 123.914900 | 47.358300 |
| 1185 | 124.466700 | 46.866700 | 1186 | 126.966700 | 47.466700 | 1187 | 126.550000 | 48.250000 | 1188 | 128.366700 | 49.466700 |
| 1189 | 127.366700 | 49.466700 | 1190 | 125.866700 | 48.066700 | 1191 | 126.266700 | 48.066700 | 1192 | 126.166700 | 47.566700 |
| 1193 | 125.466700 | 47.866700 | 1194 | 123.266700 | 47.266700 | 1195 | 123.466700 | 46.566700 | 1196 | 123.566700 | 47.766700 |
| 1197 | 124.566700 | 47.866700 | 1198 | 124.966700 | 47.266700 | 1199 | 125.366700 | 46.066700 | 1200 | 125.166700 | 45.566700 |
| 1201 | 127.166700 | 47.166700 | 1202 | 127.566700 | 46.966700 | 1203 | 126.566700 | 46.766700 | 1204 | 126.366700 | 46.266700 |
| 1205 | 125.966700 | 47.266700 | 1206 | 126.166700 | 46.766700 | 1207 | 125.466700 | 46.566700 | 1208 | 126.066700 | 46.166700 |
| 1209 | 128.066700 | 46.966700 | 1210 | 122.794400 | 45.341700 | 1211 | 124.532200 | 45.123600 | 1212 | 123.966700 | 45.066700 |
| 1213 | 123.266700 | 45.866700 | 1214 | 122.966700 | 44.866700 | 1215 | 125.966700 | 44.966700 | 1216 | 124.750000 | 43.500000 |
| 1217 | 124.416700 | 43.366700 | 1218 | 128.266700 | 43.366700 | 1219 | 125.266700 | 44.466700 | 1220 | 125.766700 | 44.666700 |
| 1221 | 126.666700 | 44.966700 | 1222 | 123.566700 | 43.666700 | 1223 | 125.366700 | 43.366700 | 1224 | 125.666700 | 42.766700 |
| 1225 | 124.966700 | 43.166700 | 1226 | 126.166700 | 42.766700 | 1227 | 126.066700 | 42.266700 | 1228 | 125.666700 | 42.533300 |
| 1229 | 119.633300 | 41.766700 | 1230 | 121.621700 | 42.133600 | 1231 | 124.066700 | 42.766700 | 1232 | 123.266700 | 42.766700 |
| 1233 | 119.966700 | 40.766700 | 1234 | 122.666700 | 42.566700 | 1235 | 123.466700 | 42.466700 | 1236 | 123.566700 | 42.466700 |
| 1237 | 124.066700 | 42.666700 | 1238 | 124.666700 | 42.766700 | 1239 | 119.466700 | 41.366700 | 1240 | 119.866700 | 41.066700 |
| 1241 | 120.866700 | 41.966700 | 1242 | 121.266700 | 41.666700 | 1243 | 121.966700 | 41.766700 | 1244 | 122.566700 | 41.466700 |
| 1245 | 123.066700 | 40.366700 | 1246 | 124.966700 | 42.066700 | 1247 | 125.166700 | 41.766700 | 1248 | 119.933300 | 42.283300 |
| 1249 | 120.066700 | 43.716700 | 1250 | 119.033300 | 42.866700 | 1251 | 118.666700 | 43.566700 | 1252 | 121.366700 | 43.600000 |
| 1253 | 121.766700 | 42.766700 | 1254 | 121.608300 | 45.405600 | 1255 | 122.162500 | 46.043900 | 1256 | 122.440600 | 46.472200 |
| 1257 | 121.966700 | 46.166700 | 1258 | 115.300000 | 41.900000 | 1259 | 116.466700 | 42.166700 | 1260 | 114.066700 | 41.966700 |
| 1261 | 111.466700 | 41.166700 | 1262 | 112.566700 | 40.566700 | 1263 | 112.566700 | 40.966700 | 1264 | 118.066700 | 43.666700 |
| 1265 | 117.466700 | 43.266700 | 1266 | 118.666700 | 41.966700 | 1267 | 119.366700 | 41.566700 | 1268 | 119.466700 | 42.166700 |
| 1269 | 122.266700 | 43.666700 | 1270 | 119.666700 | 45.566700 | 1271 | 113.166700 | 41.466700 | 1272 | 112.766700 | 41.266700 |
| 1273 | 113.266700 | 40.966700 | 1274 | 113.266700 | 40.466700 | 1275 | 113.966700 | 40.966700 | 1276 | 111.866700 | 40.366700 |
| 1277 | 111.766700 | 39.966700 | 1278 | 111.066700 | 40.666700 | 1279 | 110.566700 | 40.566700 | 1280 | 110.066700 | 41.066700 |
| 1281 | 110.066700 | 40.366700 | 1282 | 111.166700 | 39.766700 | 1283 | 109.766700 | 39.466700 | 1284 | 109.066700 | 38.866700 |
| 1285 | 108.766700 | 39.766700 | 1286 | 108.266700 | 41.066700 | 1287 | 108.666700 | 40.766700 | 1288 | 108.566700 | 41.566700 |
| 1289 | 107.166700 | 41.566700 | 1290 | 107.066700 | 40.366700 | 1291 | 112.683300 | 36.683300 | 1292 | 112.833300 | 38.066700 |
| 1293 | 113.083300 | 35.583300 | 1294 | 114.066700 | 39.766700 | 1295 | 113.766700 | 39.666700 | 1296 | 111.566700 | 38.716700 |
| 1297 | 111.466700 | 39.466700 | 1298 | 111.166700 | 38.466700 | 1299 | 110.966700 | 37.966700 | 1300 | 112.366700 | 37.366700 |
| 1301 | 113.566700 | 37.366700 | 1302 | 113.766700 | 37.566700 | 1303 | 111.866700 | 35.766700 | 1304 | 110.866700 | 35.466700 |
| 1305 | 113.066700 | 37.966700 | 1306 | 112.566700 | 37.466700 | 1307 | 112.266700 | 37.366700 | 1308 | 111.966700 | 37.066700 |
| 1309 | 111.866700 | 36.966700 | 1310 | 113.466700 | 38.066700 | 1311 | 113.666700 | 37.866700 | 1312 | 114.166700 | 40.466700 |
| 1313 | 114.266700 | 39.466700 | 1314 | 112.966700 | 39.066700 | 1315 | 113.266700 | 38.766700 | 1316 | 111.966700 | 38.366700 |
| 1317 | 112.266700 | 39.166700 | 1318 | 111.966700 | 38.966700 | 1319 | 111.266700 | 39.366700 | 1320 | 111.166700 | 38.966700 |
| 1321 | 111.266700 | 37.866700 | 1322 | 110.866700 | 37.466700 | 1323 | 110.866700 | 37.066700 | 1324 | 110.766700 | 36.766700 |
| 1325 | 110.966700 | 36.666700 | 1326 | 111.066700 | 36.366700 | 1327 | 110.766700 | 36.166700 | 1328 | 110.966700 | 35.666700 |
| 1329 | 111.266700 | 35.466700 | 1330 | 111.266700 | 35.666700 | 1331 | 111.666700 | 35.566700 | 1332 | 111.766700 | 35.366700 |
| 1333 | 111.266700 | 35.166700 | 1334 | 111.166700 | 34.966700 | 1335 | 110.766700 | 34.766700 | 1336 | 110.866700 | 35.166700 |
| 1337 | 110.566700 | 34.966700 | 1338 | 110.766700 | 35.666700 | 1339 | 109.933300 | 37.766700 | 1340 | 110.500000 | 37.966700 |
| 1341 | 110.366700 | 37.566700 | 1342 | 108.866700 | 37.666700 | 1343 | 107.666700 | 37.566700 | 1344 | 109.266700 | 36.866700 |
| 1345 | 109.466700 | 35.766700 | 1346 | 107.866700 | 35.266700 | 1347 | 108.366700 | 35.166700 | 1348 | 109.366700 | 37.866700 |
| 1349 | 110.066700 | 37.566700 | 1350 | 110.866700 | 37.766700 | 1351 | 110.166700 | 37.466700 | 1352 | 109.766700 | 37.266700 |
| 1353 | 110.066700 | 36.966700 | 1354 | 109.966700 | 36.666700 | 1355 | 109.366700 | 36.066700 | 1356 | 109.266700 | 35.666700 |
| 1357 | 110.166700 | 36.166700 | 1358 | 109.866700 | 35.766700 | 1359 | 108.166700 | 36.866700 | 1360 | 108.066700 | 35.166700 |
| 1361 | 108.566700 | 34.966700 | 1362 | 108.166700 | 34.766700 | 1363 | 107.966700 | 34.766700 | 1364 | 106.866700 | 34.866700 |
| 1365 | 107.166700 | 34.766700 | 1366 | 107.466700 | 34.566700 | 1367 | 107.666700 | 34.466700 | 1368 | 107.966700 | 34.466700 |
| 1369 | 107.866700 | 34.266700 | 1370 | 106.566700 | 34.066700 | 1371 | 109.166700 | 35.566700 | 1372 | 108.966700 | 35.066700 |
| 1373 | 107.366700 | 36.566700 | 1374 | 108.066700 | 36.466700 | 1375 | 105.066700 | 35.633300 | 1376 | 105.266700 | 35.266700 |
| 1377 | 107.366700 | 35.366700 | 1378 | 105.766700 | 35.566700 | 1379 | 105.366700 | 34.866700 | 1380 | 108.366700 | 36.066700 |
| 1381 | 107.966700 | 35.466700 | 1382 | 108.466700 | 35.666700 | 1383 | 107.266700 | 35.666700 | 1384 | 107.066700 | 35.266700 |
| 1385 | 107.666700 | 35.066700 | 1386 | 106.066700 | 35.166700 | 1387 | 106.766700 | 35.266700 | 1388 | 104.666700 | 35.066700 |
| 1389 | 104.166700 | 35.166700 | 1390 | 103.866700 | 35.366700 | 1391 | 104.566700 | 34.966700 | 1392 | 104.066700 | 34.566700 |
| 1393 | 104.666700 | 36.466700 | 1394 | 104.166700 | 37.166700 | 1395 | 105.066700 | 36.766700 | 1396 | 105.966700 | 34.666700 |
| 1397 | 105.766700 | 34.966700 | 1398 | 104.866700 | 34.766700 | 1399 | 106.266700 | 35.066700 | 1400 | 102.966700 | 37.466700 |
| 1401 | 103.066700 | 38.666700 | 1402 | 102.766700 | 37.966700 | 1403 | 101.966700 | 38.266700 | 1404 | 103.966700 | 36.366700 |
| 1405 | 104.166700 | 35.966700 | 1406 | 103.266700 | 36.766700 | 1407 | 103.166700 | 35.666700 | 1408 | 103.766700 | 35.466700 |
| 1409 | 103.666700 | 35.566700 | 1410 | 103.466700 | 35.466700 | 1411 | 103.566700 | 35.766700 | 1412 | 102.966700 | 35.766700 |
| 1413 | 103.466700 | 34.766700 | 1414 | 103.666700 | 34.666700 | 1415 | 104.266700 | 33.866700 | 1416 | 104.366700 | 34.066700 |
| 1417 | 105.266700 | 34.266700 | 1418 | 105.366700 | 34.166700 | 1419 | 106.166700 | 33.866700 | 1420 | 106.366700 | 33.966700 |
| 1421 | 106.633300 | 35.866700 | 1422 | 107.316700 | 37.783300 | 1423 | 105.766700 | 35.966700 | 1424 | 105.666700 | 36.566700 |
| 1425 | 106.166700 | 35.666700 | 1426 | 106.366700 | 35.566700 | 1427 | 105.966700 | 36.966700 | 1428 | 106.166700 | 37.366700 |
| 1429 | 106.366700 | 37.066700 | 1430 | 106.166700 | 37.966700 | 1431 | 105.266700 | 37.566700 | 1432 | 105.766700 | 37.566700 |
| 1433 | 114.691700 | 39.816700 | 1434 | 114.766700 | 41.166700 | 1435 | 115.666700 | 41.666700 | 1436 | 116.666700 | 41.266700 |
| 1437 | 117.766700 | 42.066700 | 1438 | 113.966700 | 38.266700 | 1439 | 114.166700 | 36.766700 | 1440 | 113.966700 | 41.066700 |
| 1441 | 114.366700 | 40.666700 | 1442 | 114.166700 | 40.166700 | 1443 | 114.466700 | 40.866700 | 1444 | 115.066700 | 40.666700 |
| 1445 | 115.366700 | 40.966700 | 1446 | 115.866700 | 40.966700 | 1447 | 115.566700 | 40.466700 | 1448 | 115.266700 | 40.366700 |
| 1449 | 117.666700 | 41.366700 | 1450 | 118.666700 | 41.066700 | 1451 | 117.366700 | 40.966700 | 1452 | 117.566700 | 40.566700 |
| 1453 | 118.566700 | 40.666700 | 1454 | 118.966700 | 40.466700 | 1455 | 118.366700 | 40.166700 | 1456 | 117.966700 | 40.266700 |
| 1457 | 118.766700 | 40.066700 | 1458 | 117.966700 | 39.966700 | 1459 | 114.366700 | 38.366700 | 1460 | 114.566700 | 38.666700 |
| 1461 | 114.466700 | 37.766700 | 1462 | 114.166700 | 38.066700 | 1463 | 114.766700 | 38.366700 | 1464 | 114.566700 | 38.166700 |
| 1465 | 114.666700 | 37.966700 | 1466 | 114.366700 | 38.066700 | 1467 | 115.366700 | 39.366700 | 1468 | 115.466700 | 39.366700 |
| 1469 | 115.166700 | 38.866700 | 1470 | 114.966700 | 38.766700 | 1471 | 115.266700 | 38.766700 | 1472 | 115.766700 | 39.166700 |
| 1473 | 115.666700 | 38.966700 | 1474 | 115.466700 | 38.966700 | 1475 | 115.566700 | 38.766700 | 1476 | 115.866700 | 38.766700 |
| 1477 | 115.966700 | 38.966700 | 1478 | 116.166700 | 38.966700 | 1479 | 116.466700 | 39.166700 | 1480 | 116.666700 | 38.966700 |
| 1481 | 116.766700 | 38.766700 | 1482 | 116.366700 | 39.466700 | 1483 | 116.566700 | 39.366700 | 1484 | 117.066700 | 39.766700 |
| 1485 | 116.866700 | 39.866700 | 1486 | 115.866700 | 38.166700 | 1487 | 116.566700 | 37.966700 | 1488 | 116.166700 | 38.766700 |
| 1489 | 117.466700 | 38.366700 | 1490 | 116.166700 | 38.466700 | 1491 | 116.966700 | 38.366700 | 1492 | 116.866700 | 38.666700 |
| 1493 | 116.766700 | 37.966700 | 1494 | 116.466700 | 37.766700 | 1495 | 116.766700 | 38.066700 | 1496 | 117.266700 | 38.066700 |
| 1497 | 117.166700 | 38.066700 | 1498 | 117.666700 | 38.166700 | 1499 | 114.566700 | 37.466700 | 1500 | 114.666700 | 37.366700 |
| 1501 | 114.766700 | 37.166700 | 1502 | 114.766700 | 37.066700 | 1503 | 114.966700 | 37.666700 | 1504 | 115.066700 | 37.266700 |
| 1505 | 115.066700 | 37.066700 | 1506 | 115.166700 | 37.066700 | 1507 | 115.366700 | 37.066700 | 1508 | 115.666700 | 37.066700 |
| 1509 | 115.566700 | 36.966700 | 1510 | 114.566700 | 36.966700 | 1511 | 115.466700 | 37.366700 | 1512 | 114.466700 | 36.466700 |
| 1513 | 114.566700 | 36.366700 | 1514 | 114.666700 | 36.366700 | 1515 | 115.266700 | 36.366700 | 1516 | 114.966700 | 36.466700 |
| 1517 | 114.966700 | 36.866700 | 1518 | 114.866700 | 36.966700 | 1519 | 115.266700 | 36.866700 | 1520 | 115.466700 | 36.566700 |
| 1521 | 115.066700 | 36.566700 | 1522 | 116.366700 | 35.066700 | 1523 | 117.566700 | 36.766700 | 1524 | 117.090000 | 36.710000 |
| 1525 | 116.766700 | 36.566700 | 1526 | 116.466700 | 36.266700 | 1527 | 117.266700 | 36.966700 | 1528 | 117.166700 | 37.166700 |
| 1529 | 118.366700 | 36.866700 | 1530 | 117.966700 | 36.966700 | 1531 | 117.866700 | 37.166700 | 1532 | 117.966700 | 36.466700 |
| 1533 | 118.166700 | 36.266700 | 1534 | 117.266700 | 35.166700 | 1535 | 117.366700 | 34.866700 | 1536 | 118.466700 | 37.066700 |
| 1537 | 118.366700 | 37.566700 | 1538 | 118.666700 | 37.666700 | 1539 | 119.966700 | 37.166700 | 1540 | 120.466700 | 37.366700 |
| 1541 | 120.866700 | 37.366700 | 1542 | 121.266700 | 36.866700 | 1543 | 119.266700 | 36.466700 | 1544 | 119.466700 | 36.866700 |
| 1545 | 119.466700 | 35.966700 | 1546 | 118.766700 | 36.966700 | 1547 | 118.566700 | 36.566700 | 1548 | 118.866700 | 36.666700 |
| 1549 | 116.966700 | 35.566700 | 1550 | 116.966700 | 35.466700 | 1551 | 116.366700 | 35.466700 | 1552 | 117.166700 | 34.866700 |
| 1553 | 116.666700 | 34.966700 | 1554 | 116.166700 | 35.766700 | 1555 | 117.366700 | 35.666700 | 1556 | 116.466700 | 35.966700 |
| 1557 | 116.866700 | 35.766700 | 1558 | 117.866700 | 35.966700 | 1559 | 122.066700 | 37.266700 | 1560 | 122.466700 | 37.166700 |
| 1561 | 121.666700 | 36.966700 | 1562 | 116.330000 | 37.340000 | 1563 | 117.266700 | 37.766700 | 1564 | 116.666700 | 36.966700 |
| 1565 | 116.666700 | 37.366700 | 1566 | 116.866700 | 37.666700 | 1567 | 117.466700 | 37.766700 | 1568 | 116.966700 | 37.266700 |
| 1569 | 116.466700 | 37.166700 | 1570 | 116.066700 | 36.966700 | 1571 | 116.166700 | 37.166700 | 1572 | 116.166700 | 36.566700 |
| 1573 | 116.366700 | 36.366700 | 1574 | 115.566700 | 36.566700 | 1575 | 115.766700 | 36.266700 | 1576 | 115.866700 | 36.166700 |
| 1577 | 115.966700 | 36.466700 | 1578 | 118.066700 | 37.366700 | 1579 | 117.866700 | 36.966700 | 1580 | 117.666700 | 37.566700 |
| 1581 | 117.566700 | 37.566700 | 1582 | 117.666700 | 37.866700 | 1583 | 118.166700 | 37.166700 | 1584 | 115.566700 | 35.266700 |
| 1585 | 115.566700 | 34.866700 | 1586 | 115.666700 | 35.166700 | 1587 | 115.966700 | 34.966700 | 1588 | 116.066700 | 34.866700 |
| 1589 | 115.966700 | 35.366700 | 1590 | 115.966700 | 35.566700 | 1591 | 115.566700 | 35.566700 | 1592 | 115.166700 | 35.366700 |
| 1593 | 114.366700 | 36.100000 | 1594 | 111.066700 | 34.066700 | 1595 | 112.866700 | 34.166700 | 1596 | 114.266700 | 35.666700 |
| 1597 | 114.566700 | 35.666700 | 1598 | 114.466700 | 35.966700 | 1599 | 114.566700 | 35.566700 | 1600 | 114.866700 | 35.966700 |
| 1601 | 113.966700 | 36.166700 | 1602 | 113.666700 | 35.266700 | 1603 | 113.766700 | 35.366700 | 1604 | 114.266700 | 35.266700 |
| 1605 | 114.466700 | 35.066700 | 1606 | 114.766700 | 35.166700 | 1607 | 114.166700 | 35.466700 | 1608 | 113.866700 | 35.566700 |
| 1609 | 114.766700 | 34.766700 | 1610 | 114.466700 | 34.766700 | 1611 | 114.866700 | 34.566700 | 1612 | 114.566700 | 34.566700 |
| 1613 | 114.266700 | 34.566700 | 1614 | 112.866700 | 34.766700 | 1615 | 112.566700 | 34.966700 | 1616 | 112.266700 | 34.866700 |
| 1617 | 111.666700 | 33.866700 | 1618 | 112.066700 | 34.266700 | 1619 | 112.466700 | 34.166700 | 1620 | 112.266700 | 34.566700 |
| 1621 | 111.766700 | 34.466700 | 1622 | 113.066700 | 33.966700 | 1623 | 113.266700 | 33.966700 | 1624 | 112.966700 | 33.766700 |
| 1625 | 113.366700 | 33.666700 | 1626 | 113.466700 | 33.366700 | 1627 | 113.566700 | 33.966700 | 1628 | 113.866700 | 34.066700 |
| 1629 | 114.166700 | 34.166700 | 1630 | 113.966700 | 33.866700 | 1631 | 113.666700 | 33.566700 | 1632 | 114.066700 | 33.666700 |
| 1633 | 114.066700 | 33.366700 | 1634 | 114.366700 | 33.266700 | 1635 | 114.666700 | 33.066700 | 1636 | 114.466700 | 32.766700 |
| 1637 | 114.066700 | 32.966700 | 1638 | 113.566700 | 32.766700 | 1639 | 114.366700 | 33.066700 | 1640 | 114.966700 | 32.866700 |
| 1641 | 115.466700 | 32.566700 | 1642 | 114.766700 | 32.366700 | 1643 | 115.066700 | 32.166700 | 1644 | 114.966700 | 32.066700 |
| 1645 | 115.766700 | 32.166700 | 1646 | 115.566700 | 31.866700 | 1647 | 114.566700 | 32.266700 | 1648 | 112.966700 | 33.266700 |
| 1649 | 112.966700 | 33.066700 | 1650 | 112.866700 | 32.766700 | 1651 | 112.466700 | 32.566700 | 1652 | 113.466700 | 32.466700 |
| 1653 | 112.266700 | 33.066700 | 1654 | 111.966700 | 33.066700 | 1655 | 111.566700 | 33.166700 | 1656 | 112.166700 | 32.766700 |
| 1657 | 116.366700 | 33.966700 | 1658 | 115.066700 | 34.466700 | 1659 | 116.166700 | 34.466700 | 1660 | 115.366700 | 34.166700 |
| 1661 | 115.366700 | 34.466700 | 1662 | 115.266700 | 34.666700 | 1663 | 115.166700 | 34.466700 | 1664 | 114.366700 | 34.366700 |
| 1665 | 115.566700 | 33.966700 | 1666 | 115.266700 | 33.766700 | 1667 | 114.966700 | 33.766700 | 1668 | 115.166700 | 33.466700 |
| 1669 | 114.966700 | 33.466700 | 1670 | 116.866700 | 40.366700 | 1671 | 116.666700 | 40.366700 | 1672 | 117.166700 | 40.166700 |
| 1673 | 116.366700 | 39.966700 | 1674 | 117.466700 | 40.066700 | 1675 | 117.366700 | 39.666700 | 1676 | 117.066700 | 39.466700 |
| 1677 | 117.566700 | 39.366700 | 1678 | 116.866700 | 38.966700 | 1679 | 103.266700 | 26.466700 | 1680 | 103.766700 | 27.366700 |
| 1681 | 103.566700 | 27.166700 | 1682 | 102.966700 | 26.966700 | 1683 | 104.966700 | 27.466700 | 1684 | 104.166700 | 27.666700 |
| 1685 | 104.966700 | 27.866700 | 1686 | 103.966700 | 27.766700 | 1687 | 103.766700 | 28.066700 | 1688 | 103.966700 | 28.566700 |
| 1689 | 104.266700 | 28.066700 | 1690 | 104.466700 | 28.666700 | 1691 | 103.266700 | 26.166700 | 1692 | 102.466700 | 25.966700 |
| 1693 | 103.266700 | 25.666700 | 1694 | 104.166700 | 26.266700 | 1695 | 104.366700 | 25.766700 | 1696 | 104.366700 | 24.966700 |
| 1697 | 103.966700 | 24.966700 | 1698 | 103.766700 | 25.066700 | 1699 | 103.666700 | 25.466700 | 1700 | 103.866700 | 25.666700 |
| 1701 | 99.966700 | 26.166700 | 1702 | 99.866700 | 26.566700 | 1703 | 100.266700 | 26.866700 | 1704 | 106.066700 | 27.066700 |
| 1705 | 105.666700 | 27.166700 | 1706 | 104.766700 | 27.166700 | 1707 | 106.366700 | 27.466700 | 1708 | 105.866700 | 26.866700 |
| 1709 | 105.466700 | 26.766700 | 1710 | 104.366700 | 26.866700 | 1711 | 106.366700 | 28.366700 | 1712 | 106.466700 | 27.866700 |
| 1713 | 106.866700 | 28.166700 | 1714 | 107.166700 | 27.966700 | 1715 | 107.566700 | 28.566700 | 1716 | 107.666700 | 28.866700 |
| 1717 | 107.966700 | 28.766700 | 1718 | 107.766700 | 27.966700 | 1719 | 107.566700 | 27.766700 | 1720 | 107.966700 | 27.266700 |
| 1721 | 105.766700 | 26.266700 | 1722 | 105.866700 | 26.066700 | 1723 | 105.666700 | 25.966700 | 1724 | 106.166700 | 25.766700 |
| 1725 | 104.666700 | 25.666700 | 1726 | 104.966700 | 26.166700 | 1727 | 105.566700 | 26.266700 | 1728 | 105.266700 | 25.466700 |
| 1729 | 105.066700 | 25.866700 | 1730 | 105.266700 | 25.866700 | 1731 | 105.666700 | 25.466700 | 1732 | 106.166700 | 25.166700 |
| 1733 | 105.866700 | 24.966700 | 1734 | 105.566700 | 25.166700 | 1735 | 102.866700 | 28.066700 | 1736 | 102.866700 | 27.766700 |
| 1737 | 102.566700 | 27.666700 | 1738 | 103.266700 | 27.766700 | 1739 | 103.666700 | 27.966700 | 1740 | 103.166700 | 28.366700 |
| 1741 | 102.866700 | 28.966700 | 1742 | 102.566700 | 28.666700 | 1743 | 102.466700 | 28.366700 | 1744 | 102.266700 | 28.566700 |
| 1745 | 101.566700 | 27.466700 | 1746 | 102.266700 | 27.466700 | 1747 | 102.366700 | 26.766700 | 1748 | 102.666700 | 26.666700 |
| 1749 | 102.866700 | 26.966700 | 1750 | 102.866700 | 30.066700 | 1751 | 102.866700 | 30.366700 | 1752 | 102.966700 | 30.266700 |
| 1753 | 102.866700 | 29.866700 | 1754 | 102.466700 | 29.266700 | 1755 | 102.266700 | 29.966700 | 1756 | 101.866700 | 30.766700 |
| 1757 | 101.666700 | 29.066700 | 1758 | 100.966700 | 30.066700 | 1759 | 101.166700 | 31.066700 | 1760 | 100.766700 | 31.466700 |
| 1761 | 99.966700 | 31.666700 | 1762 | 100.266700 | 30.966700 | 1763 | 98.966700 | 31.266700 | 1764 | 98.666700 | 31.866700 |
| 1765 | 97.666700 | 32.966700 | 1766 | 100.366700 | 32.366700 | 1767 | 101.066700 | 32.366700 | 1768 | 101.966700 | 31.666700 |
| 1769 | 102.366700 | 31.066700 | 1770 | 103.566700 | 32.666700 | 1771 | 103.966700 | 33.266700 | 1772 | 103.066700 | 32.166700 |
| 1773 | 102.266700 | 31.966700 | 1774 | 103.766700 | 31.666700 | 1775 | 103.266700 | 31.466700 | 1776 | 103.666700 | 31.566700 |
| 1777 | 104.566700 | 31.966700 | 1778 | 104.566700 | 32.466700 | 1779 | 105.266700 | 32.566700 | 1780 | 106.366700 | 32.266700 |
| 1781 | 105.966700 | 32.666700 | 1782 | 103.866700 | 29.866700 | 1783 | 104.266700 | 29.966700 | 1784 | 103.466700 | 29.966700 |
| 1785 | 103.566700 | 30.066700 | 1786 | 104.566700 | 28.066700 | 1787 | 104.866700 | 28.066700 | 1788 | 105.166700 | 28.266700 |
| 1789 | 105.566700 | 28.166700 | 1790 | 105.866700 | 28.066700 | 1791 | 107.266700 | 31.966700 | 1792 | 106.966700 | 32.066700 |
| 1793 | 107.166700 | 31.566700 | 1794 | 108.066700 | 32.066700 | 1795 | 107.766700 | 31.366700 | 1796 | 107.866700 | 31.166700 |
| 1797 | 107.266700 | 30.766700 | 1798 | 106.966700 | 30.866700 | 1799 | 106.966700 | 30.466700 | 1800 | 106.466700 | 30.566700 |
| 1801 | 106.366700 | 30.366700 | 1802 | 106.766700 | 30.466700 | 1803 | 104.366700 | 31.566700 | 1804 | 105.066700 | 31.766700 |
| 1805 | 105.466700 | 31.366700 | 1806 | 105.066700 | 31.166700 | 1807 | 105.366700 | 30.966700 | 1808 | 105.766700 | 30.866700 |
| 1809 | 105.266700 | 30.666700 | 1810 | 108.666700 | 31.966700 | 1811 | 109.366700 | 31.466700 | 1812 | 109.866700 | 31.066700 |
| 1813 | 109.566700 | 31.066700 | 1814 | 108.866700 | 30.966700 | 1815 | 108.466700 | 31.166700 | 1816 | 108.466700 | 30.866700 |
| 1817 | 107.866700 | 30.666700 | 1818 | 108.066700 | 30.366700 | 1819 | 108.166700 | 29.966700 | 1820 | 107.766700 | 29.866700 |
| 1821 | 107.466700 | 30.366700 | 1822 | 107.366700 | 29.766700 | 1823 | 107.166700 | 29.166700 | 1824 | 107.766700 | 29.366700 |
| 1825 | 108.166700 | 29.266700 | 1826 | 108.766700 | 28.866700 | 1827 | 108.966700 | 28.466700 | 1828 | 106.766700 | 28.966700 |
| 1829 | 106.366700 | 29.266700 | 1830 | 105.966700 | 29.366700 | 1831 | 106.366700 | 29.966700 | 1832 | 106.066700 | 29.866700 |
| 1833 | 105.766700 | 29.766700 | 1834 | 106.266700 | 29.566700 | 1835 | 105.666700 | 29.466700 | 1836 | 105.866700 | 30.066700 |
| 1837 | 102.466700 | 36.466700 | 1838 | 102.866700 | 36.366700 | 1839 | 102.066700 | 36.866700 | 1840 | 102.266700 | 36.166700 |
| 1841 | 102.566700 | 35.866700 | 1842 | 101.466700 | 36.066700 | 1843 | 101.966700 | 35.966700 | 1844 | 102.066700 | 35.566700 |
| 1845 | 101.566700 | 35.066700 | 1846 | 101.666700 | 34.766700 | 1847 | 100.666700 | 36.266700 | 1848 | 100.666700 | 35.266700 |
| 1849 | 99.966700 | 35.566700 | 1850 | 100.766700 | 35.666700 | 1851 | 100.266700 | 34.466700 | 1852 | 100.766700 | 32.966700 |
| 1853 | 81.566700 | 43.966700 | 1854 | 80.866700 | 44.066700 | 1855 | 82.866700 | 43.566700 | 1856 | 82.166700 | 43.466700 |
| 1857 | 81.166700 | 43.166700 | 1858 | 81.966700 | 43.266700 | 1859 | 82.466700 | 43.866700 | 1860 | 81.266700 | 43.766700 |
| 1861 | 83.666700 | 46.566700 | 1862 | 83.666700 | 45.966700 | 1863 | 82.966700 | 46.266700 | 1864 | 85.066700 | 46.766700 |
| 1865 | 86.266700 | 44.266700 | 1866 | 86.966700 | 44.166700 | 1867 | 87.966700 | 44.166700 | 1868 | 89.166700 | 44.066700 |
| 1869 | 89.666700 | 44.066700 | 1870 | 90.466700 | 43.866700 | 1871 | 86.366700 | 42.366700 | 1872 | 86.966700 | 42.266700 |
| 1873 | 86.766700 | 42.066700 | 1874 | 86.566700 | 42.066700 | 1875 | 82.966700 | 41.766700 | 1876 | 81.966700 | 41.466700 |
| 1877 | 82.566700 | 41.566700 | 1878 | 81.966700 | 41.866700 | 1879 | 110.466700 | 33.066700 | 1880 | 109.766700 | 32.366700 |
| 1881 | 110.866700 | 32.966700 | 1882 | 110.766700 | 32.066700 | 1883 | 111.566700 | 32.266700 | 1884 | 111.566700 | 32.566700 |
| 1885 | 110.666700 | 31.766700 | 1886 | 111.666700 | 31.166700 | 1887 | 111.866700 | 31.766700 | 1888 | 110.766700 | 31.366700 |
| 1889 | 110.966700 | 30.966700 | 1890 | 110.866700 | 30.566700 | 1891 | 110.666700 | 30.266700 | 1892 | 115.966700 | 31.666700 |
| 1893 | 116.266700 | 32.266700 | 1894 | 116.666700 | 31.466700 | 1895 | 116.366700 | 31.466700 | 1896 | 116.366700 | 30.866700 |
| 1897 | 116.566700 | 30.666700 | 1898 | 116.366700 | 30.466700 | 1899 | 116.166700 | 30.166700 | 1900 | 117.266700 | 30.766700 |
| 1901 | 116.966700 | 30.066700 | 1902 | 117.466700 | 30.266700 | 1903 | 117.866700 | 30.666700 | 1904 | 109.466700 | 29.066700 |
| 1905 | 109.666700 | 27.966700 | 1906 | 109.466700 | 28.566700 | 1907 | 109.666700 | 28.666700 | 1908 | 109.966700 | 28.666700 |
| 1909 | 110.166700 | 28.266700 | 1910 | 109.166700 | 27.366700 | 1911 | 109.766700 | 27.466700 | 1912 | 109.866700 | 27.866700 |
| 1913 | 109.666700 | 26.566700 | 1914 | 109.766700 | 26.266700 | 1915 | 110.366700 | 26.366700 | 1916 | 110.266700 | 26.566700 |
| 1917 | 110.866700 | 26.466700 | 1918 | 110.666700 | 26.766700 | 1919 | 110.566700 | 27.066700 | 1920 | 110.966700 | 27.166700 |
| 1921 | 111.266700 | 27.766700 | 1922 | 111.266700 | 28.366700 | 1923 | 111.466700 | 28.866700 | 1924 | 111.366700 | 29.666700 |
| 1925 | 111.166700 | 29.466700 | 1926 | 110.266700 | 29.566700 | 1927 | 111.566700 | 24.966700 | 1928 | 111.366700 | 25.066700 |
| 1929 | 112.266700 | 25.766700 | 1930 | 112.566700 | 25.366700 | 1931 | 113.966700 | 25.766700 | 1932 | 113.666700 | 25.566700 |
| 1933 | 113.366700 | 26.666700 | 1934 | 113.566700 | 26.766700 | 1935 | 113.766700 | 26.566700 | 1936 | 112.866700 | 27.266700 |
| 1937 | 112.966700 | 27.066700 | 1938 | 112.166700 | 26.766700 | 1939 | 112.566700 | 26.466700 | 1940 | 112.866700 | 26.266700 |
| 1941 | 112.266700 | 27.466700 | 1942 | 112.066700 | 27.666700 | 1943 | 111.466700 | 27.666700 | 1944 | 111.466700 | 27.366700 |
| 1945 | 111.266700 | 27.066700 | 1946 | 111.366700 | 26.466700 | 1947 | 111.666700 | 25.966700 | 1948 | 111.966700 | 25.666700 |

**Table S2.** Explanation of environmental variables.

| **Environment variable** | **Description** | **Unit** |
| --- | --- | --- |
| bio1 | Annual Mean Temperature | °C |
| bio2 | Mean diurnal temperature range | °C |
| bio3 | Isothermality (Bio2/Bio7) (×100) | - |
| bio4 | Seasonal variation coefficient of temperature (standard deviation×100) | - |
| bio5 | Max Temperature of Warmest Month |  |
| bio6 | Min Temperature of Coldest Month | °C |
| bio7 | Temperature Annual Range | °C |
| bio8 | Mean temperature of the wettest quarter | °C |
| bio9 | Mean Temperature of Driest Quarter | °C |
| bio10 | Mean Temperature of Warmest Quarter | °C |
| bio11 | Mean Temperature of Coldest Quarter | °C |
| bio12 | Annual Precipitation | mm yr⁻¹ |
| bio13 | Precipitation of Wettest Month | mm |
| bio14 | Precipitation of Driest Month | mm |
| bio15 | Precipitation Seasonality | mm |
| bio16 | Precipitation of Wettest Quarter | mm |
| bio17 | Precipitation of the Driest Quarter | mm |
| bio18 | Precipitation of Warmest Quarter | mm quarter⁻¹ |
| bio19 | Precipitation of Coldest Quarter | mm quarter⁻¹ |
| uvb1 | Annual Mean UV-B | J m⁻² day⁻¹ |
| uvb2 | UV-B Seasonality | J m⁻² day⁻¹ |
| uvb3 | Mean UV-B of lightest Month | J m⁻² day⁻¹ |
| uvb4 | Mean UV-B of Lowest Month | J m⁻² day⁻¹ |
| elev | Elevation | m |
| aspect | Aspect | - |
| slope | Slope | ° |
| t_bulk | Topsoil bulk density | g cm⁻³ |
| t_cecsoil | Topsoil cation exchange capacity of the soil | kg⁻¹ soil |
| t_cecclay | Topsoil cation exchange capacity of the clay fraction | kg⁻¹ clay |
| t_clay | Topsoil clay fraction | % |
| t_ece | Topsoil salinity | dS m⁻¹ |
| t_esp | Topsoil sodicity | % |
| t_gravel | Topsoil gravel content | % |
| t_oc | Topsoil organic carbon | % weight |
| t_ph | Topsoil pH | - |
| t_refbulk | Topsoil reference bulk density | g cm⁻³ |
| t_sand | Topsoil sand fraction | % |
| t_silt | Topsoil silt fraction | % |
| t_texture | Topsoil texture class | - |
| t_usda | Topsoil USDA texture class | - |

Note: The variables isothermality (bio3), temperature seasonality (bio4), Topsoil texture class (t_texture) and Aspect are expressed as dimensionless indices or percentages. Given their nature as ratios or standardized scales, they are presented. without physical units in this table.

**Table S3.** Evaluation metrics of MaxEnt model generated by ENMeval

| **Parameter Settings** | **FC** | **RM** | **Delta.AICc** | **AUC.DIFF** | **OR10** | **AUC** | **TSS** |
| --- | --- | --- | --- | --- | --- | --- | --- |
| Default | LQHPT | 1.0 | 36.1061 | 0.0512 | 0.2539 | 0.837±0.012 | 0.797±0.020 |
| Optimized | LH | 0.5 | 0 | 0.0093 | 0.1193 | 0.962±0.009 | 0.878±0.025 |

FC, feature combination; RM regularization multiplier.

**Table S4.** Validation performance of individual models.

| **Algorithm** | **KAPPA** | **ROC** | **TSS** |
| --- | --- | --- | --- |
| CTA | 0.427 ± 0.043 | 0.797 ± 0.016 | 0.456 ± 0.038 |
| FDA | 0.435 ± 0.023 | 0.807 ± 0.009 | 0.461 ± 0.020 |
| GBM | 0.486 ± 0.020 | 0.846 ± 0.010 | 0.539 ± 0.028 |
| GLM | 0.488 ± 0.023 | 0.839 ± 0.011 | 0.543 ± 0.022 |
| MARS | 0.492 ± 0.018 | 0.848 ± 0.010 | 0.551 ± 0.012 |
| MAXENT | 0.504 ± 0.019 | 0.859 ± 0.011 | 0.558 ± 0.017 |
| RF | 0.511 ± 0.028 | 0.867 ± 0.012 | 0.518 ± 0.027 |
| XGBOOST | 0.492 ± 0.030 | 0.853 ± 0.011 | 0.543 ± 0.026 |

Note: Values are mean ± SD based on validation results across pseudo-absence and model runs. ROC, receiver operating characteristic; TSS, true skill statistic.

**Table S5.** Validation performance of ensemble models.

| **Filtering metric** | **Ensemble algorithm** | **ROC** | **TSS** |
| --- | --- | --- | --- |
| TSS | EMmean | 0.863 ± 0.011 | 0.580 ± 0.023 |
| TSS | EMca | 0.839 ± 0.014 | 0.574 ± 0.022 |
| TSS | EMwmean | 0.863 ± 0.011 | 0.581 ± 0.022 |
| ROC | EMmean | 0.863 ± 0.009 | 0.583 ± 0.018 |
| ROC | EMca | 0.844 ± 0.011 | 0.576 ± 0.019 |
| ROC | EMwmean | 0.863 ± 0.010 | 0.582 ± 0.019 |

Note: ROC, receiver operating characteristic; TSS, true skill statistic. EMmean, ensemble mean; EMca, committee averaging; EMwmean, weighted mean ensemble.

**Table S6.** Centroid migration routes under different SSP scenarios based on EMwmeanByTSS.

| **SSP scenario** | **Period** | **Latitude (°N)** | **Longitude (°E)** | **Stepwise shift (km)** | **Cumulative shift (km)** | **Net displacement (km)** |
| --- | --- | --- | --- | --- | --- | --- |
| Current | Current | 35.32 | 113.47 | 0.00 | 0.00 | 0.00 |
| SSP1-2.6 | 2030s | 35.59 | 113.50 | 30.05 | 30.05 | 29.98 |
| SSP1-2.6 | 2050s | 35.76 | 113.38 | 22.42 | 52.47 | 49.70 |
| SSP1-2.6 | 2070s | 36.04 | 113.23 | 33.72 | 86.20 | 82.74 |
| SSP1-2.6 | 2090s | 36.30 | 113.11 | 30.68 | 116.88 | 113.33 |
| SSP2-4.5 | 2030s | 35.88 | 113.21 | 66.45 | 66.45 | 66.34 |
| SSP2-4.5 | 2050s | 36.49 | 112.46 | 94.81 | 161.26 | 157.90 |
| SSP2-4.5 | 2070s | 36.52 | 112.70 | 21.40 | 182.66 | 149.75 |
| SSP2-4.5 | 2090s | 36.88 | 112.16 | 62.61 | 245.27 | 209.14 |
| SSP3-7.0 | 2030s | 35.69 | 113.20 | 47.69 | 47.69 | 47.65 |
| SSP3-7.0 | 2050s | 35.90 | 112.78 | 44.54 | 92.23 | 89.79 |
| SSP3-7.0 | 2070s | 36.76 | 112.45 | 99.21 | 191.44 | 183.63 |
| SSP3-7.0 | 2090s | 37.29 | 112.59 | 61.01 | 252.45 | 232.79 |
| SSP5-8.5 | 2030s | 36.11 | 113.07 | 94.65 | 94.65 | 94.50 |
| SSP5-8.5 | 2050s | 36.38 | 112.71 | 44.29 | 138.94 | 136.03 |
| SSP5-8.5 | 2070s | 36.88 | 112.52 | 57.85 | 196.79 | 192.66 |
| SSP5-8.5 | 2090s | 37.08 | 112.79 | 32.45 | 229.24 | 204.05 |

Note: Stepwise shift is the centroid distance between consecutive time slices within the same SSP pathway. Cumulative shift is the sum of stepwise shifts from the historical baseline. Net displacement is the great-circle distance from the historical baseline centroid to the centroid of each future period.
